# Wealth-based inequality in the continuum of maternal health service utilisation in 16 sub-Saharan African countries

**DOI:** 10.1186/s12939-023-02015-0

**Published:** 2023-10-02

**Authors:** Anteneh Asefa, Samson Gebremedhin, Tiara Marthias, Herfina Nababan, Aliki Christou, Aline Semaan, Aduragbemi Banke-Thomas, Hanani Tabana, Fadhlun M. Alwy Al-beity, Jean-Paul Dossou, Keneni Gutema, Thérèse Delvaux, Catherine Birabwa, Mardieh Dennis, Fassou Mathias Grovogui, Barbara McPake, Lenka Beňová

**Affiliations:** 1 Department of Public Health, Institute of Tropical Medicine, Antwerp, Belgium; 2 School of Public Health, Addis Ababa University, Addis Ababa, Ethiopia; 3 School of Population and Global Health, Nossal Institute for Global Health, The University of Melbourne, Melbourne, Australia; 4 Department of Health Policy and Management, Faculty of Medicine, Public Health, and Nursing, Universitas Gadjah Mada, Yogyakarta, Indonesia; 5 Institut für Public Health und Pflegeforschung, Universität Bremen, Bremen, Germany; 6 School of Human Sciences, University of Greenwich, London, UK; 7 Faculty of Epidemiology and Population Health, London School of Hygiene and Tropical Medicine, London, UK; 8 School of Public Health, University of the Western Cape, Cape Town, South Africa; 9 Department of Obstetrcis/Gynaecology, Muhimbili University of Health and Allied Sciences, Dar es Salaam, Tanzania; 10 Centre de Recherche en Reproduction Humaine Et en Démographie, Cotonou, Bénin; 11 School of Public Health, College of Medicine and Health Sciences, Hawassa University, Hawassa, Ethiopia; 12 Makerere University School of Public Health, Kampala, Uganda; 13 Last Mile Health, Monrovia, Liberia; 14 Centre National de Formation Et de Recherche en Santé Rurale (CNFRSR), Maferinyah, Forécariah Guinea

**Keywords:** Antenatal care, Demographic and health survey, Facility-based childbirth, Inequality, Maternal continuum of care, Postnatal care, Sub-Saharan Africa

## Abstract

**Background:**

Persistent inequalities in coverage of maternal health services in sub-Saharan Africa (SSA), a region home to two-thirds of global maternal deaths in 2017, poses a challenge for countries to achieve the Sustainable Development Goal (SDG) targets. This study assesses wealth-based inequalities in coverage of maternal continuum of care in 16 SSA countries with the objective of informing targeted policies to ensure maternal health equity in the region.

**Methods:**

We conducted a secondary analysis of Demographic and Health Survey (DHS) data from 16 SSA countries (Angola, Benin, Burundi, Cameroon, Ethiopia, Gambia, Guinea, Liberia, Malawi, Mali, Nigeria, Sierra Leone, South Africa, Tanzania, Uganda, and Zambia). A total of 133,709 women aged 15-49 years who reported a live birth in the five years preceding the survey were included. We defined and measured completion of maternal continuum of care as having had at least one antenatal care (ANC) visit, birth in a health facility, and postnatal care (PNC) by a skilled provider within two days of birth. We used concentration index analysis to measure wealth-based inequality in maternal continuum of care and conducted decomposition analysis to estimate the contributions of sociodemographic and obstetric factors to the observed inequality.

**Results:**

The percentage of women who had 1) at least one ANC visit was lowest in Ethiopia (62.3%) and highest in Burundi (99.2%), 2) birth in a health facility was less than 50% in Ethiopia and Nigeria, and 3) PNC within two days was less than 50% in eight countries (Angola, Burundi, Ethiopia, Gambia, Guinea, Malawi, Nigeria, and Tanzania). Completion of maternal continuum of care was highest in South Africa (81.4%) and below 50% in nine of the 16 countries (Angola, Burundi, Ethiopia, Guinea, Malawi, Mali, Nigeria, Tanzania, and Uganda), the lowest being in Ethiopia (12.5%). There was pro-rich wealth-based inequality in maternal continuum of care in all 16 countries, the lowest in South Africa and Liberia (concentration index = 0.04) and the highest in Nigeria (concentration index = 0.34). Our decomposition analysis showed that in 15 of the 16 countries, wealth index was the largest contributor to inequality in primary maternal continuum of care. In Malawi, geographical region was the largest contributor.

**Conclusions:**

Addressing the coverage gap in maternal continuum of care in SSA using multidimensional and people-centred approaches remains a key strategy needed to realise the SDG3. The pro-rich wealth-based inequalities observed show that bespoke pro-poor or population-wide approaches are needed.

**Supplementary Information:**

The online version contains supplementary material available at 10.1186/s12939-023-02015-0.

## Introduction

In the last two decades, significant progress has been made toward improving maternal and child health outcomes, particularly in low- and middle-income countries (LMICs) [1]. Globally, maternal mortality ratio declined by 34.3% between 2000 and 2020 [1] and neonatal mortality rate declined 42% between 1990 and 2015 [2]. However, the burden of poor maternal and child health outcomes remains high in many LMICs. Sub-Saharan Africa (SSA) in particular was estimated to account for 70% of global maternal deaths in 2020, and children in the region are 14 times more likely to die before reaching their fifth birthday than those in high-income countries [3].

Large within-country socioeconomic and geographic inequalities in coverage of maternal and newborn health interventions exist in SSA [4–6]. For example, lower coverage of antenatal care (ANC) and facility-based childbirth have been reported among the poor in Cameroon, Ethiopia, Madagascar, Uganda, Zambia, and Zimbabwe [5]. Concurrently, with increasing population-level coverage, the inequality gaps were widening over time in three of these countries (Cameroon, Madagascar, and Uganda) [5]. The persistent poverty-related inequalities in coverage and outcomes of maternal and child health pose a challenge for countries to achieve the Sustainable Development Goal (SDG) targets and in the long term, will impede the achievement of universal health coverage (UHC) [7, 8]. Previous studies have also documented that wealth-based inequality is a major contributor to inequitable access to maternal and newborn health interventions in SSA [2, 3, 9–11], the highest inequality being in the West African region [11]. Wealth status also affects accessibility to essential health services and is closely linked with other key determinants of health such as education [3–6]. A study commissioned by the Countdown to 2030 for Women’s, Children’s and Adolescents’ Health indicated that eliminating within-country wealth-related inequalities in 36 SSA countries could increase the composite coverage of reproductive, maternal, newborn and child health services by 10 to 20 percentage points [11]. And thus, designing interventions that alleviate such inequalities and are tailored towards the most-deprived populations is essential for achieving SDG 3.

Within the context of maternal and child health interventions aimed at reducing preventable maternal and child deaths [12–15], completion of care along the continuum of maternal care has been shown to directly improve not only maternal health outcomes, but also early neonatal outcomes and long term childhood development [16–19]. However, a recent pooled analysis of Demographic and Health Surveys (DHS) of 33 SSA countries reported that the level of completion of maternal continuum of care (at least one ANC visit and birth in a health facility and postnatal care (PNC) visit within six weeks of childbirth) was only 36% [20]. Generating evidence on the level of wealth-based inequalities in the continuum of maternal care in SSA—currently underresearched—plays a critical role in designing and implementing targeted policies to reduce the iniquitous enrolment into and drop out from the continuum of maternal health services in the region. Existing evidence on wealth-based inequalities in the coverage of maternal health services in the region lacks depth to depict inequalities in the completion of maternal continuum of care—a key indicator which can reveal iniquitous coverage of retention in care—and that may result in the underestimation of maternal and newborn health gains which could be achieved by eliminating wealth-based inequalities.

In this paper, we reflect on inequalities in completion of maternal continuum of care vis-à-vis countries’ health system performance in maternal and child health which we believe is key to identifying and prioritising areas of improvement for evidence-based policy making [21]. Comparative health system performance analysis across countries in a particular region allows for contextual benchmarking, cross-country learning, and advocating for effective political leadership. Further, there is also a need to continuously monitor the progress toward equitable access to and utilisation of key maternal and child health services in regions with high maternal and child mortality burden.

This study aims to fill the gap in the literature by (1) estimating country-by-country coverage of ANC, facility-based childbirth and PNC and comparing these across countries; (2) measuring wealth-based inequalities in coverage of maternal continuum of care and; (3) determining factors contributing to the inequalities by using the most recent nationally-representative surveys from 16 SSA countries. To our knowledge, this is the first study that comprehensively assesses wealth-based inequalities in 16 sub-Saharan African countries using the maternal continuum of care approach during pregnancy, childbirth, and the postnatal period. The findings of this study will contribute to the knowledge base in the field which could be used by countries to prioritise context-oriented policies and strategies to increase the utilisation of maternal health services and reduce wealth-based inequality in completion of maternal continuum of care.

## Methods

### Study design and participants

**Fig. 1:**
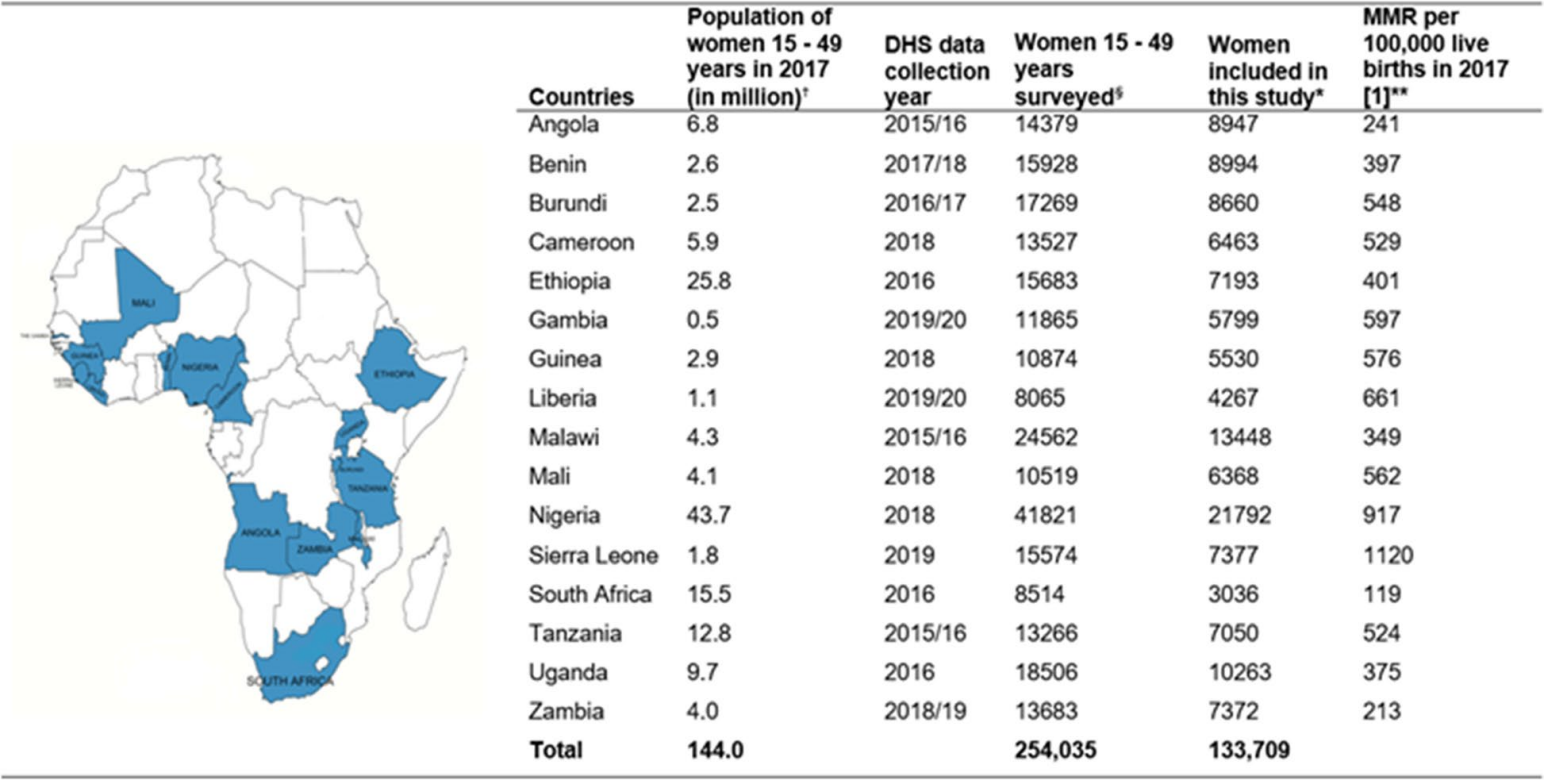
Countries included in this study and DHS sample size

This study is based on secondary analysis of standard DHS data from 16 countries in SSA (Angola, Benin, Burundi, Cameroon, Ethiopia, Gambia, Guinea, Liberia, Malawi, Mali, Nigeria, Sierra Leone, South Africa, Tanzania, Uganda, and Zambia). The countries were selected based on the criterion of having a recent Standard DHS (conducted between 2015 and the time of the analysis in 2022). The earliest DHS data was from Tanzania (2015/16) and the most recent was from the Gambia (2019/20) (Fig. 1). The DHSs are nationally representative cross-sectional household surveys gathering data on key health indicators. DHS participants are selected using a two-stage cluster sampling technique from administrative regions stratified by type of place of residence (urban/rural). These administrative regions have predefined enumeration areas (EAs) or clusters. A sample of EAs are then selected using probability proportionate to size in each stratum (urban or rural) from the list of the predefined EAs. Then, members—all women aged 15–49 years and men aged 15–59 years—of households sampled in EAs selected using a systematic random sampling method were surveyed. Standard DHSs have large sample sizes to allow adequate power to estimate key health indicators in a population. In this study, we used individual women’s datasets (individual recode) and included women aged 15—49 years who reported a livebirth in the five years preceding the survey. For women who gave birth more than once in the five-year recall period, the most recent birth was considered in this study as key maternity care variables (e.g. ANC) are only collected for women’s most recent birth. The inclusion of only recent births within the last five-year recall period was made to minimise the chance of recall bias and mixing of information (between two or more pregnancy experiences) if women are asked about their experiences of maternal health service utilization during their last two or more pregnancies in the five-year period; additionally, data on key variables, mainly ANC, are only collected for the most recent birth in the recall period. A total of 133,709 women were included in the study with each country having a sample size between 3,036 (South Africa) and 21,792 (Nigeria) (Fig. 1). Data from each country were analysed separately.

### Indicators and definitions

#### Antenatal care

Receipt of ANC was defined as a woman reporting any facility-based ANC visit during the pregnancy of the most recent live birth. We categorised this indicator into two: 1) women who had at least one ANC visit (categorised as “yes – those who had one or more ANC visits” or “no – those who had no ANC visit”), and 2) women who had at least four ANC visits (categorised as “yes – those who had four or more ANC visits” or “no – those who had no ANC visit or those who had one to three ANC visits”) (Additional file 1.A). We chose at least four ANC visits in line with WHO’s focused ANC recommendations [22] which was in use during the five-year recall periods covered by the DHSs and international indicators designed to track coverage of ANC [23]. We considered women who responded “do not know” to the number of ANC visits question, as having one ANC visit based on the assumption that women are less likely to forget if they had at least one ANC visit. This question was preceded by the question “did you have ANC visit?” with a yes/no response option and women who said yes to the question were subsequently asked about the number of visits [24].

#### Facility-based childbirth

Women who reported having given birth in a health facility, regardless of the type and ownership of the facility, were considered as having had facility-based childbirth (Additional file 1.A). Women who responded “other” to the question on place of childbirth were considered as having given birth outside health facility with the assumption that 1) women are less likely to forget if they gave childbirth in health facilities, and 2) all possible health facility types in a country were included in the DHS questionnaire used in that particular country.

#### Postnatal care within two days of birth by a skilled provider

Women who reported receiving a PNC for themselves, in a health facility or at home by a skilled provider, within the first 48 h of birth were considered as having received PNC within two days (Additional file 1.A). We defined this indicator in line with recommended current and previous WHO guidelines [25, 26] and international indicators designed to track coverage of PNC [27]. List of skilled providers by country is provided in Additional file 1.B.

#### Maternal continuum of care

The outcome variable of this study is completion of maternal continuum of care, a composite variable, categorised into primary and secondary outcome variables, each with binary (yes or no) options. The main outcome variable is “primary maternal continuum of care”, defined as having had at least one ANC visit AND birth in a health facility AND PNC by a skilled provider within two days of birth. If any or all of these three components were not received, the continuum of care was considered not complete. Accordingly, no weight was applied to any of the defining variables (ANC, facility-based childbirth, and PNC). The secondary outcome variable is “secondary maternal continuum of care”, defined as having had at least four ANC visits AND birth in a health facility AND PNC within the first two days of birth. We used four ANC visits as a requirement to secondary maternal continuum of care to align with the World Health Organization’s focused ANC approach [22] which was implemented during most or all of the five-year recall periods of the DHSs and international indicators to track progress toward the SDGs [23].

#### Wealth index

Living standard of women’s households computed by the DHS program based on ownership of selected assets and stratified in quintiles as poorest, poorer, medium, richer, and richest. We used the country-specific and urban/rural adjusted wealth quintiles variable (v190a) designed by the DHS team, because the normal wealth index variable (v190) is largely urban in its construction and fails to identify the poorest of the poor as is the case in most SSA countries where a substantial portion of the population is rural [28].

#### Age group

Age of women in completed years at the time of the most recent live birth categorised in seven categories of a 5-year interval each (15–19, 20–24, 25–29, 30–34, 35–39, 40–44, and 45–49).

#### Marital status

Women’s relationship status at the time of survey categorised as in union or not in union.

#### Educational status

Highest educational attainment of women at the time of survey categorised as no education, primary education, secondary education, or higher education.

#### Place of residence

Type of the place where the participant was surveyed categorised by the DHS as urban or rural.

#### Parity

The number of total children a woman gave birth to at the time of the pregnancy preceding the most recent live birth, categorised into 5 groups (0, 1–2, 3–4, 5–6, and > 7).

#### Region

Administrative regions within a country as used by the DHSs.

### Data analysis

Data for all countries were analysed separately using StataSE v.16 (StataCorp, College Station, Texas, United States). To account for the complexity and multi-stage cluster sampling design of the DHS, we used weighted data analysis, based on women’s individual sample weights. We also adjusted our analyses for clustering and stratification using the svyset Stata command. In the datasets of all countries included in this study, there were no missing values for the variables which were used to construct the three key maternal health variables (ANC, facility-based childbirth, and PNC) which were used to define maternal continuum of care. We used percentages and frequency distributions to present sociodemographic characteristics of the analysis sample (age group, educational status, place of residence, wealth quintile, and parity) and the percentages of women utilising the three maternal health services (ANC, birth in a health facility, and PNC). To check for drop offs of women in the maternal continuum of care, we also presented the percentage point difference between 1) the percentage of at least four and the percentage of at least one ANC visits, 2) the percentage of at least one ANC visit and the percentage of facility-based childbirth, and 2) the percentage of facility-based childbirth and the percentage of PNC within two days for each country.

To estimate absolute inequality in maternal continuum of care across wealth quintiles (poorest, poorer, middle, richer, and richest), we applied the Erreygers normalised concentration index analyses [29, 30] using the *conindex* Stata command [31] accounting for clustering; we presented concentration indices with their corresponding 95% confidence intervals (95% CI) and concentration curves. The Erreygers concentration index—*E(h)*—is specially designed for estimating absolute inequalities in outcome variables with binary (yes/no) responses; the formula is displayed below [29].

$$E\left(h\right)=\frac{8}{{n}^{2}\left({b}_{h}-{a}_{h}\right)}\sum_{i=1}^{n}{z}_{i}{h}_{i}$$

where “h” is the health variable; “n” is number of individuals in the study; “b_h_” is the upper bound of the health variable; “a_h_” is the lower bound of the health variable; and “z” derivative of the socioeconomic rank of individuals*.*

The concentration index ranges between + 1 and -1; a positive concentration index (> 0) indicates a higher concentration of the outcome variable (completed maternal continuum of care) among the wealthier quintiles. On the other hand, a negative concentration index (< 0) indicates a higher concentration of the outcome variable among the poorer quintiles.

To quantify the observed wealth-based inequality in maternal continuum of care contributed by sociodemographic and regional factors in each country, we also conducted decomposition analyses using the modified Wagstaff decomposition method [32]; the formula is displayed below.

$$\mathrm{CCI}={\sum }_{k=1}^{K}{C}_{k}\left({\beta }_{k}{\overline{\mathrm{Z}} }_{k}/\mu h\right)+\left({GC}_{\varepsilon }/\mu h\right)$$

where C_k_ is the concentration index of the k^th^ contributing factor, β_k_ is a coefficient derived from linear equation of the contributing factors, $\overline{Z }$_k_ is mean of the k^th^ contributing factor, GCε is the generalized concentration index of the error factor (residual component).

As the primary outcome variable of our study is binary, we applied the generalised linear model with a binomial family and probit link in the decomposition analysis [33]. In addition to wealth quintiles, based on existing evidence on contributors to health inequalities [11, 34], we also included six independent variables (contributing factors) in the model—age group, marital status, place of residence, educational status, parity, and region within country.

## Results

### Sociodemographic characteristics of study sample

The mean age of participants at the time of the most recent birth was highest in Burundi (29.1 years) and lowest in Tanzania (25.5 years); the highest proportions of women were in the age group 20-24 years in nine of the 16 countries included in the analysis (Additional file 2). Urban residents accounted for the majority of the respondents in the Gambia (66.8%), Angola (64.1%), South Africa (64.0%), and Liberia (56.4%), whereas rural residents accounted for the majority of the respondents in the remaining 12 countries, the highest being in Burundi (90.2%), followed by Ethiopia (87.2%) and Malawi (85.7%). The proportion of participants who did not attend formal education ranged from 1.4% in South Africa to 75.9% in Guinea. Median parity at the time of index pregnancy was 1 in Cameroon and South Africa, 3 in Nigeria, and 2 in the remaining 13 countries.

### Utilisation of maternal health services

#### Antenatal care

The percentage of women who had at least one ANC visit was lowest in Ethiopia (62.3%) followed by Nigeria (74.0%), and highest in Burundi (99.2%). Less than half of the women included in the analysis had at least four ANC visits in Ethiopia (31.8%), Guinea (35.3%), Mali (43.3%), and Burundi (49.3%); Liberia had the highest (87.3%) proportion of women who had at least four ANC visits followed by Sierra Leone (78.6%). The difference between the percentage of women who had at least one ANC visit and those who had four visits was highest in Guinea (50.7%) followed by Burundi (50.0%), Malawi (47.6%), and Tanzania (47.3%). The difference was lowest in Liberia (10.6%) (Table 1).

**Table 1:**
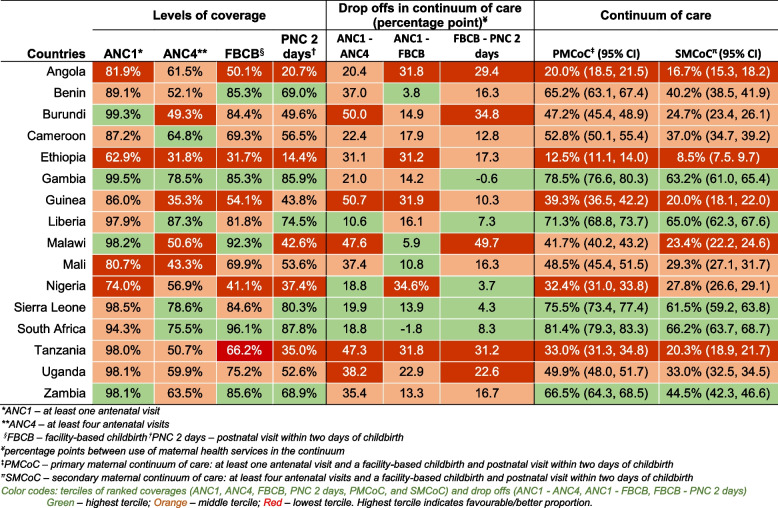
Coverage of maternal health services and gaps in maternal continuum of care by country

#### Facility-based childbirth

The percentage of births which took place in health facilities was more than 80% in eight of the 16 countries, the highest being in South Africa (96.1%) (Table 1). In two countries, fewer than half of the women reported giving birth in a facility: Nigeria (41.1%) and Ethiopia (31.7%). In South Africa, the percentage of women who gave birth in a facility was higher than the percentage of women who had at least one ANC visit (94.3%), whereas it was the opposite in the remaining 15 countries. The difference between the percentage of women who had at least one ANC visit and the percentage of women who had facility-based childbirth was more than 25 percentage points in five countries (Nigeria, Guinea, Angola, Tanzania, and Ethiopia) and more than 10 percentage points in 13 of the 16 countries.

#### Postnatal care

In seven of the 16 countries, fewer than half of the women received PNC within two days of birth, the least in Ethiopia (14.4%) followed by Angola (20.7%) (Table 1). The utilisation of PNC was more than 80% in South Africa, the Gambia, and Sierra Leone. The drop from the percentages of women who had a facility-based childbirth and those who received PNC was steepest in Malawi (49.7) followed by Burundi (34.8), Tanzania (31.2), and Angola (29.4). The difference was negligible in the Gambia (-0.6) and very low in in Nigeria (3.7) and Sierra Leone (4.3).

#### Maternal continuum of care

The percentage of women who completed the primary maternal continuum of care was highest in South Africa (81.4%) and the Gambia (78.5%) and lowest in Ethiopia (12.5%); primary maternal continuum of care was higher than 50% in seven of the 16 countries (Cameroon, Benin, Zambia, Liberia, Sierra Leone, Gambia, South Africa; Table 1). The completion of secondary maternal continuum of care and the ranking of countries was also similar, ranging from 8.5% in Ethiopia to 66.2% in South Africa (Table 1).

#### Wealth-based inequality in the continuum of maternal care

**Fig. 2:**
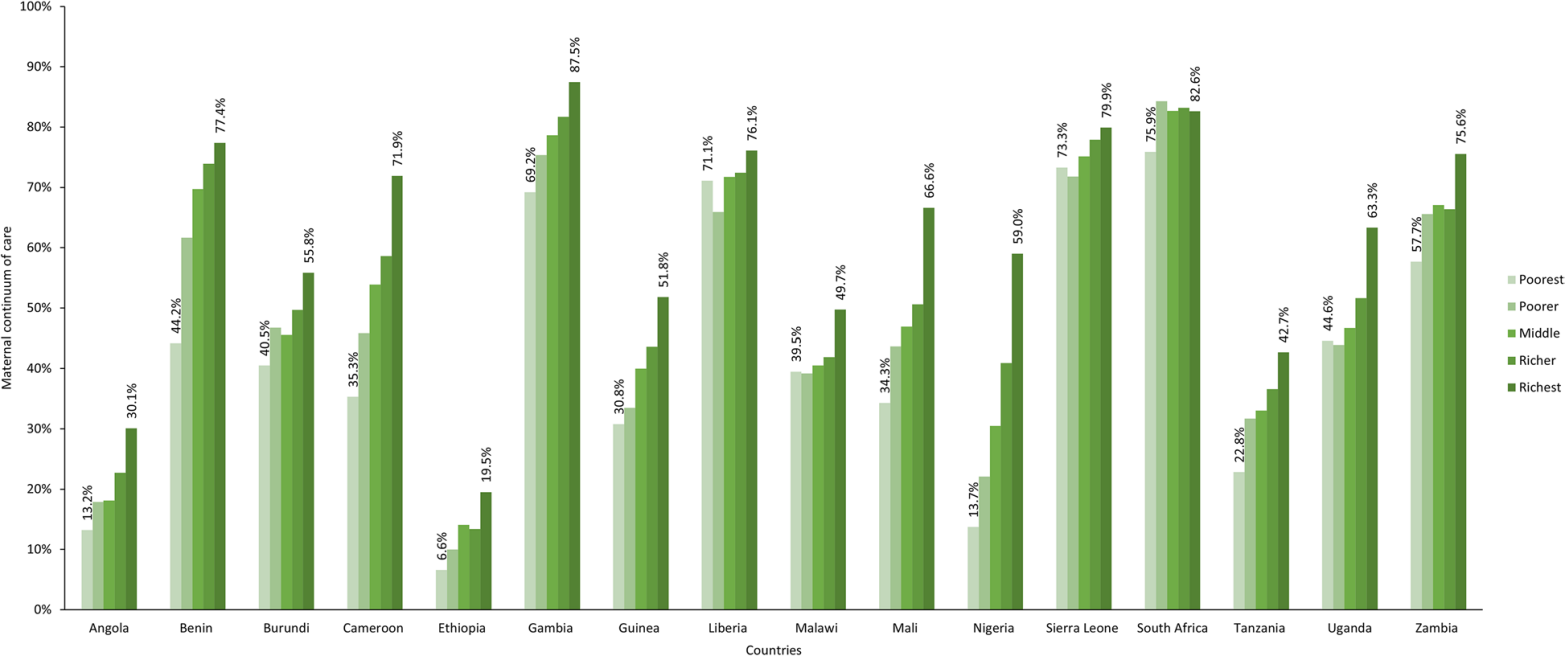
Percentage of women completing primary maternal continuum of care, by wealth index, by country

**Fig. 3:**
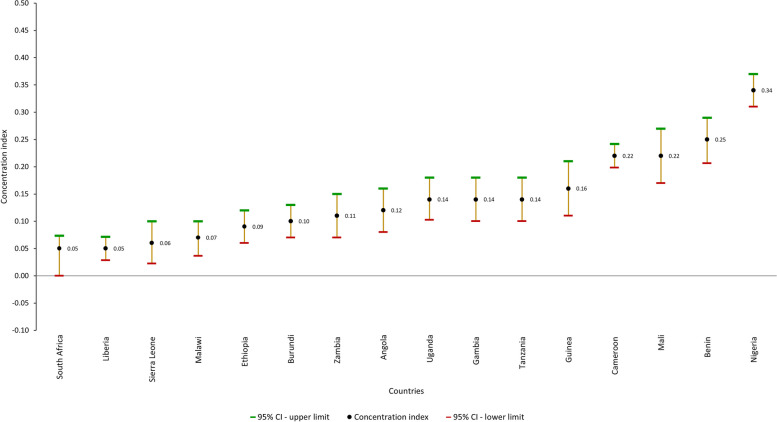
Concentration indices of primary maternal continuum of care across countries (0 = equality)

As shown in Fig. 2, the level of primary maternal continuum of care was highest among women in the richest household quintile in all countries except South Africa, where coverage did not follow a progressive pattern. The results of the concentration index analyses are displayed in Fig. 3. There was statistically significant pro-rich inequality in the primary maternal continuum of care in all 16 countries. The concentration indices ranged from 0.05 in South Africa and Liberia (least unequal) to 0.34 in Nigeria (most unequal); the second-highest inequality was in Benin (0.25), followed by Mali and Cameroon (0.22). Concentration indices were less than 0.1 in five countries, indicating a relatively equitable coverage of continuum of maternal care (Fig. 3). The concentration curves for primary maternal continuum of care for all countries are displayed in Additional file 3. We also calculated concentration indices to estimate wealth-based inequalities for secondary maternal continuum of care and found that pro-rich inequality existed in all 16 countries—the three countries with the highest (most unequal) concentration indices were Nigeria (0.31), Cameroon (0.28), and Benin (0.27) (Additional file 4).

#### Decomposition of inequalities in completion of maternal continuum of care

The decomposition analysis showed that wealth index was the largest contributor to inequality in primary maternal continuum of care in all countries except Malawi, where administrative region was the highest contributor (Additional file 5). Educational status was the second largest contributor to inequality in primary maternal continuum of care (higher coverage among more educated women) in seven countries (Burundi, Cameroon, Gambia, Mali, Nigeria, Tanzania, and Zambia) whereas it was administrative region in six countries (Angola, Benin, Ethiopia, Liberia, Sierra Leone, and Uganda), Malawi (age group), and Tanzania (parity). In Guinea and South Africa, place of residence was the second largest contributor to inequality in primary maternal continuum of care. Place of residence had negative elasticity in all countries, indicating that women from rural areas were less likely to complete the primary maternal continuum of care (Additional file 5). Similarly, parity at the time of index pregnancy had negative elasticity in all countries except Liberia, indicating that the higher the parity, the less likely women were to complete primary maternal continuum of care. On the other hand, marital status had positive elasticity in all countries except Guinea, indicating that women in union were more likely to complete primary maternal continuum of care.

## Discussion

We found that while high levels of ANC1 visits were achieved across most countries included in this study, there were significant coverage gaps between ANC1 and ANC4 visits with the largest gap as high as 50.7 percentage points (Guinea). Even in South Africa—the best-performing country—the gap reached 18.8 percentage points. In general, facility-based childbirth and PNC also had lower coverage than ANC. Consequently, we observed low coverage of primary maternal continuum of care, with only seven of the 16 countries (Cameroon, Benin, Zambia, Liberia, Sierra Leone, Gambia, South Africa) achieving coverage levels above 50%. The study also highlights pro-rich inequality in completing the maternal continuum of care, with varying degrees of inequalities across the 16 countries. The coverage of both primary and secondary maternal continuum of care that we estimated might be lower than those estimated by other studies which used PNC use within six weeks postpartum instead of two days as part of the definition of maternal continuum of care [20]. We preferred to use the two days postpartum cut-off point as majority of maternal deaths in the postpartum period—which could be largely prevented by essential early PNC—occur during the first two days of birth [25]. Further, the decomposition analysis showed that the inequalities were mainly attributed to women’s wealth status (15 countries) followed by educational status (seven countries) and administrative region (six countries). Here, we reflect on our study findings to describe why key maternal health indicators of coverage were high or low in the included countries to inform maternal health interventions in low-resource settings.

We observed narrower gaps between the poorest and the wealthiest in countries with either very low or very high overall coverage levels. This is in line with previous findings [35, 36]: moderate levels of maternal health services are most associated with high levels of inequality. In most cases, women in the richest group will utilise services initially available and opportunities for poorer groups to access services increase as coverage expands. As wealth and education level are highly correlated, especially in determining utilisation of maternal health services in low-resource settings [37], we also found similar disparity patterns in coverage levels across women’s education groups. On the other hand, we found a comparable level of high coverage and low wealth-based inequality in maternal continuum of care in Sierra Leone (low-income country) and South Africa (upper-middle-income country). This could be due to strategies such as the Free Health Care Initiative in Sierra Leone which was launched in 2010 and substantially increased the utilisation of maternal health services by the poor and decreased wealth-based inequalities in the coverage of maternal continuum of care [38]. However, such initiatives should be supported by strategies which will curb service delivery challenges due to increased demand for services, stockouts, and staff shortages to prevent backsliding of gains made [39].

The large drop offs along the maternal continuum of care indicate that women were not receiving the optimal benefit from evidence-based interventions. This is in line with previous studies that also highlight the coverage gaps between various maternal and child health services in LMICs, where coverage of the maternal continuum of care remained low regardless of very high ANC4 coverage [4, 40, 41]. Despite growing evidence suggesting that progress has been made towards maternal health-related goals of the SDGs, such levels of drop offs could hinder efforts to attain UHC by 2030 [34, 42]. For example, despite gradual progress in key maternal health indicators in Tanzania, fragmented and intermittent implementation of high-impact interventions including quality improvement guidelines—due to reliance on donor financing among others—remained a key challenge to improving maternal continuum of care [43]. Countries with higher ANC1 coverage but lower ANC4 coverage (Burundi, Malawi, and Guinea), could learn from countries with lower ANC1 coverage but higher ANC4 (Angola, Nigeria, and Cameroon) on how to improve retention in ANC through strengthening the quality of ANC services [44].

The large drop offs along the maternal continuum of care also indicate missed opportunities to maintain the uptake of maternal health services before, during, and after childbirth. For instance, women who gave birth in a health facility are more likely to receive PNC services from the same facility, before getting discharged, if they have a positive childbirth experience [45]. However, several countries such as Malawi, Burundi, Tanzania, Angola, and Uganda have considerable differences in the coverage levels between facility-based childbirth and timely PNC. Therefore, there is a need to further investigate and address the barriers to continuum of care—including experiences of care—in such settings. Additionally, trust in the skills of traditional birth attendants, cultural beliefs, and poor quality of care have been documented as barriers to access and use of life-saving interventions along the continuum of care for maternal health in Africa [46–48]. However, ensuring the maternal continuum of care is not a guarantee to avert maternal deaths from preventable causes without high-quality care. In the Gambia, Liberia, and Sierra Leone—three of the five countries with high levels of coverage of primary maternal continuum of care—the maternal mortality ratio per 100,000 live births was 600 or higher in 2017; other countries with lower coverage of primary maternal continuum of care had substantially lower maternal mortality ratios [9].

Interventions to reduce maternal health service coverage gaps should consider wealth-related inequality. In countries with high wealth-based inequality, such as Mali, Cameroon, Benin, and Nigeria, a targeted intervention approach is needed for populations in lower wealth quintiles. Mapping poverty status and prioritising poor and remote communities, through pro-poor publicly funded or subsidised health services is one approach which has been successful in improving health service coverage in other LMICs such as Peru, Brazil, and Bangladesh [49–51]. Other approaches could include the provision of free or reduced-fee health services to the poor [52], incentives for health workers to work in underserved communities [53], task-shifting of maternal health services [54], or establishing cash-transfer programs [55, 56] to incentivise women to use health services. A whole-population approach may work in countries with low national coverage of maternal continuum of care and a significantly higher coverage in the wealthiest population group. This type of approach could be appropriate for countries such as Angola and Ethiopia. In predominantly agrarian societies such as those of Ethiopia, addressing health system bottlenecks in the implementation of successful close-to-community programs—such as the health extension program—is imperative to improve maternal continuum of care [57]. Concurrently, embedding context-oriented monitoring and accountability frameworks for maternal continuum of care in countries’ health systems is instrumental to improve maternal continuum of care, track progress, and learn from implementation [58].

Our decomposition analysis showed which salient factors contributed to the inequalities in maternal continuum of care and significant reduction in wealth inequality could reduce these inequalities. Reducing extreme poverty in some of the countries included in this study could contribute to more equitable retention in the maternal continuum of care, particularly in countries such as Angola, Sierra Leone, Nigeria, and Tanzania where poverty levels are above 30% [59]. On the other hand, rising levels of income and wealth inequality will widen socioeconomic inequalities in maternal continuum of care. For instance, Angola’s Gini index, a measure of inequality, rose from 47.8 to 51.3 between 2015 and 2018 [60]. Future studies in other high maternal mortality settings such as South Asia should also prioritise decomposing wealth-based inequalities in the maternal continuum of care to quantify the contribution of other socioeconomic and obstetric characteristics and put forward actionable and context-oriented recommendations.

Financial protection plays a key role in achieving UHC, including for maternal health. Accordingly, countries with very low health insurance coverage, low health care use, and high out-of-pocket payments such as Angola, Burundi, Cameroon, Nigeria, and Uganda must revisit their financing of maternal health services [61]. Most countries in SSA have implemented social security schemes, including in Ethiopia (Productive Safety Nets Programme), Malawi (Social Cash Transfer Scheme), Namibia (Government Institutions Pension Fund), Zambia (Kalomo Pilot), Zimbabwe (National Social Protection Policy framework), among others [62–64]. However, only few schemes cover workers in the informal public sector and the schemes that exist tend to be limited to basic services. Countries should therefore consider expanding a system that guarantees access to quality health services and financial protection regardless of the contributory status and increasing the benefit package to cover transportation cost and other indirect costs that hinder timely use of maternal health services [46, 65, 66]. Strengthening maternal referral systems, particularly in countries where most women reside in rural and remote areas, could also be used to promote integrated care management that ensures coverage across the maternal continuum of care [67–69].

### Strengths and limitations

As we did not pool data from multiple countries during analysis, our study provides an opportunity to compare wealth-based inequalities in maternal continuum of care across 16 SSA countries and reflect on key country-specific health system interventions proven to have resulted in better equity in maternal health. Additionally, as we used Erreygers concentration index analysis, which is specifically designed for binary outcome variables, our findings provide the closest estimates of inequalities in maternal continuum of care in the 16 SSA countries. However, this study has several caveats. First, the nature of household survey data which relied on self-reporting poses possible recall bias that could affect the measurement of variables included in this study. Second, to maintain comparability across countries, we could not include some quality performance indicators. For instance, we did not include quality-adjusted clinical components antenatal, intrapartum or postnatal care to estimate effective coverage of these services due to lack of consistent measurement of these key indicators across countries. Future studies could consider the measurement of inequality in effective coverage of maternal continuum of care by combining household self-reported information on the experience of maternal care with facility-based data such as level of service readiness and clinical competence of health workers. Third, the inclusion of only the most recent birth in the five-year recall period could have led to the under-representation of the wealth-based inequalities which would have been observed if multiple experiences of women with short birth intervals (less than five years) were included. Fourth, as wealth quintile is relative, a participant’s lifestyle in the “poorest” quintile in one country may not be the same as that of a participant in the “poorest” quintile in another country, which will make comparisons of coverage of services by wealth index across countries challenging. Fifrth, due to lack of data on morbidities among women of reproductive age group in the DHS, we could not account for the possible role of morbidity on the (increased) uptake of maternal health services, especially in countries with high levels of sexually transmitted diseases including HIV, gestational diabetes, and hypertension among the population of pregnant women.

## Conclusion

Despite recognition of its importance and numerous efforts targeting improvement, maternal continuum of care remains low in many SSA countries, with nine of the 16 countries estimated to have less than 50% coverage; only four of the remaining seven countries had a coverage of 70% or more. Addressing the coverage gap using multidimensional and people-centred approaches remains a key strategy needed to realise UHC and the SDG3. The varying profile of low use among the poor observed across the countries shows that bespoke approaches that could either target the poorest or apply across the population are needed in different countries.

### Supplementary Information

**Additional file 1: Table S1.** A. categories of key variables, B. Skilled providers for provision of postnatal care at the time of the survey, by country.

**Additional file 2: Table S2.** Sociodemographic characteristics of participants, by country.

**Additional file 3: Figure S1.** Concentration curves for primary maternal continuum of care.

**Additional file 4: Figure S2.** Concentration indices of secondary maternal continuum of care (0 – line of equality).

**Additional file 5: Table S3.** Decomposition analysis of inequality in primary maternal continuum of care.

## Data Availability

Data from the DHS are available on request for research purposes at www.DHSprogram.com.

## Abbreviations

ANC: Antenatal care
DHS: Demographic and Health Survey
EA: Enumeration area
LMICs: Low- and middle-income countries
PNC: Postnatal care
SDG: Sustainable Development Goal
SSA: Sub-Saharan Africa
UHC: Universal health coverage
