# Supplementary material for: Wealth-based inequality in the continuum of maternal health service utilisation in 16 sub-Saharan African countries
Source: Int J Equity Health. 2023 Oct 2;22:203. doi: 10.1186/s12939-023-02015-0 (PMC10544383; doi:10.1186/s12939-023-02015-0)
Supplement: Supplementary file 1 — Additional file 1: Table S1. A. categories of key variables, B. Skilled providers for provision of postnatal care at the time of the survey, by country. [file 12939_2023_2015_MOESM1_ESM.docx]

**Additional File 1**

**A. categories of key variables**

| **Variable** | **Categories** | **Description** |
| --- | --- | --- |
| At least one antenatal care visit | 1. Yes | Women who had one or more antenatal care visit/s |
|  | 1. No | Women who did not have any antenatal care visit |
| At least four antenatal care visits | 1. Yes | Women who had four or more antenatal care visits |
|  | 1. No | Women who did not have any antenatal care visit  Women who had one to three antenatal care visit/s |
| Facility-based childbirth | 1. Yes | Women who gave birth in health facilities as per the definitions in the DHS of respective countries |
|  | 1. No | Women who gave birth outside health facilities as per the definitions in the DHS of respective countries. “Other” responses in the question assessing place of delivery/childbirth were also considered as having birth outside a health facility |
| Postnatal care within two days of birth by a skilled provider | 1. Yes | Women who received PNC in a health facility or at home by a skilled provider, within the first 48 hours of birth |
|  | 1. No | Women who did not receive PNC in a health facility or at home by a skilled provider  Women who received PNC in a health facility or at home by a skilled provider after the first 48 hours of birth |

**B. Skilled providers for provision of postnatal care at the time of the survey, by country**

| **Country** | **Skilled providers** | **Non-skilled providers** |
| --- | --- | --- |
| Angola | - Doctor - Nurse - Midwife | - Birth attendant - Traditional birth attendant |
| Benin | - Doctor - Nurse - Auxiliary nurse - Head nurse - Midwife | - Traditional birth attendant - Community / village health worker - Other |
| Burundi | - Doctor - Nurse - Midwife | - Traditional birth attendant - Community / village health worker - Other |
| Cameroon | - Doctor - Nurse - Midwife - Auxiliary midwife | - Traditional birth attendant - Community / village health worker |
| Ethiopia | - Doctor - Nurse - Midwife - Health officer | - Health extension - Other |
| Gambia | - Doctor - Nurse - Midwife - Auxiliary midwife / community nurse attendant | - Community / village health worker - Other |
| Guinea | - Doctor - Nurse - Midwife - Technical health officer | - Traditional birth attendant - Community / village health worker - Other |
| Liberia | - Doctor - Nurse - Midwife - Technical health officer | - Traditional birth attendant - Community / village health worker - Other |
| Malawi | - Doctor / clinical officer / medical assistant - Nurse - Midwife | - Patient attendant - Health surveillance assistant |
| Mali | - Doctor - Nurse - Midwife - Auxiliary midwife | - Trained traditional birth attendant - Traditional birth attendant - Community / village health worker - Relative/friend - Other |
| Nigeria | - Doctor - Nurse - Midwife - Auxiliary midwife | - Community health extension worker - Traditional birth attendant - Community / village health worker - Other |
| Sierra Leone | - Doctor - Nurse - Midwife - Auxiliary midwife | - Traditional birth attendant - Community / village health worker |
| South Africa | - Doctor / gynecologist - Nurse - Midwife | - Traditional birth attendant - Community / village health worker - Other |
| Tanzania | - Doctor / assistant medical officer - Clinical officer - Assistant clinical officer - Nurse - Assistant nurse - Midwife - Maternal and child health aide | - Community health worker - Trained traditional birth attendant - Traditional birth attendant - Other |
| Uganda | - Doctor - Nurse - Midwife - Auxiliary midwife - Nursing aide/assistant | - Traditional birth attendant - Community / village health worker - Other |
| Zambia | - Doctor - Nurse - Midwife - Clinical officer | - Community / village health assistant - Traditional birth attendant - Community / village health worker - Other |
