# Supplementary material for: Wealth-based inequality in the continuum of maternal health service utilisation in 16 sub-Saharan African countries
Source: Int J Equity Health. 2023 Oct 2;22:203. doi: 10.1186/s12939-023-02015-0 (PMC10544383; doi:10.1186/s12939-023-02015-0)
Supplement: Supplementary file 2 — Additional file 2: Table S2. Sociodemographic characteristics of participants, by country. [file 12939_2023_2015_MOESM2_ESM.docx]

**Additional file 2. Sociodemographic characteristics of participants, by country**

| **Characteristics** | **Angola** | **Benin** | **Burundi** | **Cameroon** | **Ethiopia** | **Gambia** | **Guinea** | **Liberia** | **Malawi** | **Mali** | **Nigeria** |
| --- | --- | --- | --- | --- | --- | --- | --- | --- | --- | --- | --- |
|  | (N = 8947) | (N = 8994) | (N = 8660) | (N = 7613) | (N = 7193) | (N = 5799) | (N = 5530) | (N = 4267) | (N = 13,448) | (N = 6368) | (N = 21,792) |
|  | n (%) | n (%) | n (%) | n (%) | n (%) | n (%) | n (%) | n (%) | n (%) | n (%) | n (%) |
| Age group at the time of most recent birth (years) |  |  |  |  |  |  |  |  |  |  |  |
| 15-19 | 1674 (19.7) | 1087 (12.0) | 681 (7.6) | 1110 (16.8) | 835 (11.0) | 565 (10.5) | 1006 (18.3) | 801 (19.9) | 2643 (19.6) | 1087 (16.4) | 2672 (12.2) |
| 20-24 | 2222 (26.2) | 2241 (24.8) | 2144 (24.0) | 1662 (25.1) | 1960 (25.8) | 1237 (23.0) | 1231 (22.4) | 1005 (25.0) | 3877 (28.7) | 1604 (24.2) | 5063 (23.1) |
| 25-29 | 1870 (22.0) | 2469 (27.3) | 2286 (25.6) | 1733 (26.2) | 2031 (26.8) | 1479 (27.5) | 1300 (23.7) | 915 (22.7) | 3126 (23.1) | 1542 (23.3) | 5732 (26.2) |
| 30-34 | 1248 (14.7) | 1717 (19.0) | 1949 (21.8) | 1194 (18.1) | 1437 (18.9) | 1068 (19.9) | 997 (18.2) | 611 (15.2) | 2159 (16.0) | 1238 (18.7) | 4356 (19.9) |
| 35-39 | 1033 (12.2) | 1036 (11.5) | 1198 (13.4) | 692 (10.5) | 928 (12.2) | 744 (13.9) | 610 (11.1) | 466 (11.6) | 1194 (8.8) | 816 (12.3) | 2822 (12.9) |
| 40-44 | 401 (4.7) | 413 (4.6) | 614 (6.9) | 197 (3.0) | 354 (4.7) | 243 (4.5) | 294 (5.4) | 204 (5.1) | 448 (3.3) | 299 (4.5) | 1079 (4.9) |
| 45-49 | 47 (0.6) | 69 (0.8) | 68 (0.8) | 24 (0.4) | 44 (0.6) | 35 (0.7) | 49 (0.9) | 25 (0.6) | 68 (0.5) | 37 (0.6) | 188 (0.9) |
| Mean ± SD | 26.9 ± 7.3 | 28.0 ± 6.7 | 29.1 ± 6.8 | 27.0 ± 6.7 | 28.1 ± 6.7 | 28.5 ± 6.7 | 27.5 ± 7.4 | 27.0 ± 7.3 | 26.3 ± 6.8 | 27.5 ± 7.1 | 28.3 ± 6.9 |
| Marital status at the time of the survey |  |  |  |  |  |  |  |  |  |  |  |
| Not in union | 2259 (26.6) | 682 (7.6) | 1089 (12.2) | 1314 (19.9) | 481 (6.3) | 461 (8.6) | 401 (7.3) | 1401 (34.8) | 2335 (17.3) | 265 (4.0) | 1274 (5.8) |
| In union | 6235 (73.4) | 8348 (92.4) | 7852 (87.8) | 5299 (80.1) | 7109 (93.7) | 4911 (91.4) | 5086 (92.7) | 2625 (65.2) | 11180 (82.7) | 6358 (96.0) | 20637 (94.2) |
| Place of residence |  |  |  |  |  |  |  |  |  |  |  |
| Urban | 5448 (64.1) | 3523 (39.0) | 880 (9.8) | 3626 (47.3) | 969 (12.8) | 3589 (66.8) | 1651 (30.1) | 2269 (56.4) | 1940 (14.4) | 1442 (21.8) | 8712 (39.8) |
| Rural | 3046 (35.9) | 5508 (61.0) | 8061 (90.2) | 4047 (52.7) | 6621 (87.2) | 1783 (33.2) | 3837 (69.9) | 1757 (43.6) | 11576 (85.7) | 5181 (78.2) | 13199 (60.2) |
| Educational status |  |  |  |  |  |  |  |  |  |  |  |
| No education | 2279 (26.8) | 5807 (64.3) | 4118 (46.1) | 2131 (27.8) | 4791 (63.1) | 2454 (45.7) | 4165 (75.9) | 1366 (33.9) | 1690 (12.5) | 4743 (71.6) | 9738 (44.4) |
| Primary education | 3220 (37.9) | 1650 (18.3) | 3790 (42.4) | 2400 (31.3) | 2150 (28.3) | 945 (17.6) | 612 (11.2) | 984 (24.4) | 8863 (65.6) | 805 (12.2) | 3293 (15.0) |
| Secondary education | 2731 (32.2) | 1437 (15.9) | 964 (10.8) | 2720 (35.4) | 420 (5.5) | 1717 (32) | 590 (10.8) | 1506 (37.4) | 2700 (20.0) | 978 (14.8) | 6962 (31.8) |
| Higher education | 265 (3.1) | 136 (1.5) | 69 (0.8) | 423 (5.5) | 230 (3.0) | 256 (4.8) | 120 (2.2) | 170 (4.2) | 262 (1.9) | 97 (1.5) | 1919 (8.8) |
| Wealth index |  |  |  |  |  |  |  |  |  |  |  |
| Poorest | 1803 (21.2) | 1836 (20.3) | 2023 (22.6) | 1530 (19.9) | 1609 (21.2) | 1133 (21.1) | 1212 (22.1) | 864 (21.5) | 3209 (23.7) | 1259 (19.0) | 4436 (20.2) |
| Poorer | 1791 (21.1) | 1790 (19.8) | 1906 (21.3) | 1588 (20.7) | 1599 (21.1) | 1138 (21.2) | 1139 (20.8) | 821 (20.4) | 2937 (21.7) | 1335 (20.2) | 4618 (21.1) |
| Middle | 1756 (20.7) | 1840 (20.4) | 1821 (20.4) | 1611 (21.0) | 1602 (21.1) | 1082 (20.2) | 1137 (20.7) | 856 (21.3) | 2722 (20.1) | 1387 (21.0) | 4608 (21.0) |
| Richer | 1740 (20.5) | 1825 (20.2) | 1672 (18.7) | 1522 (19.8) | 1449 (19.1) | 1018 (18.9) | 1042 (19.0) | 773 (19.2) | 2494 (18.5) | 1347 (20.3) | 4448 (20.3) |
| Richest | 1404 (16.5) | 1739 (19.3) | 1519 (17.0) | 1422 (18.5) | 1330 (17.5) | 1001 (18.6) | 957 (17.4) | 711 (17.7) | 2154 (15.9) | 1294 (19.5) | 3802 (17.4) |
| Parity at the time of index pregnancy |  |  |  |  |  |  |  |  |  |  |  |
| 0 | 1769 (20.8) | 1806 (20.0) | 1516 (17) | 2542 (33.1) | 1445 (19.1) | 1096 (20.4) | 1041 (19.0) | 1097 (27.2) | 3311 (24.5) | 1154 (17.4) | 3796 (17.3) |
| 1 -2 | 2918 (34.4) | 3125 (34.6) | 3042 (34.0) | 2291 (29.9) | 2288 (30.2) | 1826 (34.0) | 1981 (36.1) | 1419 (35.2) | 5029 (37.2) | 2026 (30.6) | 7312 (33.4) |
| 3 - 4 | 1931 (22.7) | 2265 (25.1) | 2207 (24.7) | 1559 (20.3) | 1751 (23.1) | 1301 (24.2) | 1414 (25.8) | 845 (21.0) | 3192 (23.6) | 1637 (24.7) | 5161 (23.6) |
| 5 - 6 | 1152 (13.6) | 1235 (13.7) | 1357 (15.2) | 793 (10.3) | 1225 (16.1) | 741 (13.8) | 710 (12.9) | 455 (11.3) | 1386 (10.3) | 1043 (15.8) | 3101 (14.2) |
| ≥ 7 | 725 (8.5) | 599 (6.6) | 819 (9.2) | 490 (6.4) | 880 (11.6) | 407 (7.6) | 341 (6.2) | 210 (5.2) | 598 (4.4) | 762 (11.5) | 2541 (11.6) |
| Median (Interquartile Range) | 2 (1 - 4) | 2 (1 - 4) | 2 (1 - 4) | 1 (0 - 3) | 2 (1- 5) | 2 (1 - 4) | 2 (1 - 4) | 2 (1 - 4) | 2 (1 - 3) | 2 (1 - 5) | 3 (1 - 5) |

**Additional file 2 – continued**

| **Characteristics** | **Sierra Leone** | **South Africa** | **Tanzania** | **Uganda** | **Zambia** |
| --- | --- | --- | --- | --- | --- |
|  | (N = 7377) | (N = 3036) | (N = 7050) | (N = 10,263) | (N = 7372) |
|  | n (%) | n (%) | n (%) | n (%) | n (%) |
| Age group at the time of most recent birth (in years) |  |  |  |  |  |
| 15-19 | 1204 (16.4) | 432 (14.2) | 1169 (16.5) | 1633 (16.1) | 1417 (19.4) |
| 20-24 | 1894 (25.9) | 826 (27.2) | 1815 (25.6) | 2892 (28.5) | 1943 (26.5) |
| 25-29 | 1692 (23.1) | 805 (26.5) | 1593 (22.5) | 2437 (24.0) | 1510 (20.6) |
| 30-34 | 1311 (17.9) | 569 (18.8) | 1228 (17.4) | 1613 (15.9) | 1295 (17.7) |
| 35-39 | 856 (11.7) | 294 (9.7) | 859 (12.1) | 091 (10.7) | 800 (10.9) |
| 40-44 | 306 (4.2) | 104 (3.4) | 376 (5.3) | 445 (4.4) | 330 (4.5) |
| 45-49 | 63 (0.9) | 6 (0.2) | 38 (0.5) | 42 (0.4) | 30 (0.4) |
| Mean ± SD | 27.3 ± 7.1 | 27.1 ± 6.5 | 25.5 ± 7.2 | 27.0 ± 6.9 | 26.9 ± 7.2 |
| Marital status at the time of the survey |  |  |  |  |  |
| Not in union | 1329 (18.1) | 1693 (55.8) | 1392 (19.7) | 1896 (18.7) | 1819 (24.8) |
| In union | 5997 (81.9) | 1343 (44.2) | 5687 (80.3) | 8256 (81.3) | 5506 (75.2) |
| Place of residence |  |  |  |  |  |
| Urban | 2795 (38.2) | 1942 (64.0) | 2123 (30.0) | 2346 (23.1) | 2811 (38.4) |
| Rural | 4531 (61.9) | 1094 (36.0) | 4955 (70.0) | 7807 (76.9) | 4513 (61.6) |
| Educational status |  |  |  |  |  |
| No education | 3857 (52.7) | 42 (1.4) | 1350 (19.1) | 1061 (10.5) | 689 (9.4) |
| Primary education | 1033 (14.1) | 249 (8.2) | 4580 (64.7) | 6091 (60.0) | 3595 (49.1) |
| Secondary education | 2214 (30.2) | 2394 (78.9) | 1086 (15.3) | 2285 (22.5) | 2726 (37.2) |
| Higher education | 221 (3.0) | 351 (11.6) | 63 (0.9) | 715 (7.0) | 316 (4.3) |
| Wealth index |  |  |  |  |  |
| Poorest | 1524 (20.8) | 773 (25.5) | 1526 (21.6) | 2089 (20.6) | 1679 (22.9) |
| Poorer | 1544 (21.1) | 667 (22.0) | 1484 (21.0) | 2060 (20.3) | 1589 (21.7) |
| Middle | 1485 (20.3) | 617 (20.3) | 1429 (20.2) | 2027 (20.0) | 1493 (20.4) |
| Richer | 1487 (20.3) | 523 (17.2) | 1392 (19.7) | 2033 (20.0) | 1355 (18.5) |
| Richest | 1286 (17.6) | 456 (15.0) | 1248 (17.6) | 1942 (19.1) | 1208 (16.5) |
| Parity at the time of index pregnancy |  |  |  |  |  |
| 0 | 1741 (23.8) | 1040 (34.3) | 1748 (24.7) | 2084 (20.5) | 1836 (25.1) |
| 1 -2 | 2730 (37.3) | 1586 (52.2) | 2443 (34.5) | 3417 (33.7) | 2499 (34.1) |
| 3 - 4 | 1703 (23.3) | 326 (10.7) | 1471 (20.8) | 2229 (22.0) | 1577 (21.5) |
| 5 - 6 | 812 (11.1) | 61 (2.0) | 816 (11.5) | 1284 (12.7) | 914 (12.5) |
| ≥ 7 | 340 (4.6) | 23 (0.8) | 601 (8.5) | 1138 (11.2) | 498 (6.8) |
| Median (Interquartile Range) | 2 (1 - 4) | 1 (0 - 2) | 2 (1 - 4) | 2 (1 - 4) | 2 (1 - 4) |
