# Supplementary material for: Wealth-based inequality in the continuum of maternal health service utilisation in 16 sub-Saharan African countries
Source: Int J Equity Health. 2023 Oct 2;22:203. doi: 10.1186/s12939-023-02015-0 (PMC10544383; doi:10.1186/s12939-023-02015-0)
Supplement: Supplementary file 3 — Additional file 3: Figure S1. Concentration curves for primary maternal continuum of care. [file 12939_2023_2015_MOESM3_ESM.docx]

**Additional file 3. Concentration curves for primary maternal continuum of care**

| **Angola**  **** | **Benin**  **** |
| --- | --- |
| **Burundi**  **** | **Cameroon**  **** |
| **Ethiopia**  **** | **Gambia**  **** |
| **Guinea**  **** | **Liberia**  **** |
| **Malawi**  **** | **Mali**  **** |
| **Nigeria**  **** | **Sierra Leone**  **** |
| **South Africa**  **** | **Tanzania**  **** |
| **Uganda**  **** | **Zambia**  **** |
