# Supplementary material for: Wealth-based inequality in the continuum of maternal health service utilisation in 16 sub-Saharan African countries
Source: Int J Equity Health. 2023 Oct 2;22:203. doi: 10.1186/s12939-023-02015-0 (PMC10544383; doi:10.1186/s12939-023-02015-0)
Supplement: Supplementary file 4 — Additional file 4: Figure S2. Concentration indices of secondary maternal continuum of care (0 – line of equality). [file 12939_2023_2015_MOESM4_ESM.docx]

**Additional file 4. Concentration indices of secondary maternal continuum of care (0 – line of equality)**
