# Supplementary material for: Wealth-based inequality in the continuum of maternal health service utilisation in 16 sub-Saharan African countries
Source: Int J Equity Health. 2023 Oct 2;22:203. doi: 10.1186/s12939-023-02015-0 (PMC10544383; doi:10.1186/s12939-023-02015-0)
Supplement: Supplementary file 5 — Additional file 5: Table S3. Decomposition analysis of inequality in primary maternal continuum of care. [file 12939_2023_2015_MOESM5_ESM.docx]

**Additional file 5 Decomposition analysis of inequality in primary maternal continuum of care**

| **Decomposition analysis of inequality in primary maternal continuum of care in Angola** | | | | | | |
| --- | --- | --- | --- | --- | --- | --- |
| **Characteristics** | | **Elasticity** | **Conc. Index** | **Contribution** | **Total contribution** | **% contribution** |
| **Age in years (at the time of the index birth)** | |  |  |  |  |  |
|  | < 20 | Ref |  |  |  |  |
|  | 20-24 | 0.018 | -0.001 | 0.000 | **0.001** | -0.02 |
|  | 25-29 | 0.024 | 0.038 | 0.001 |  | 0.77 |
|  | 30-34 | 0.028 | 0.022 | 0.001 |  | 0.51 |
|  | 35-39 | 0.005 | -0.020 | 0.000 |  | -0.09 |
|  | 40-44 | 0.008 | -0.012 | 0.000 |  | -0.09 |
|  | 45-49 | 0.000 | -0.005 | 0.000 |  | 0.00 |
|  | |  |  |  |  | **1.08** |
| **Marital status at the time of the survey** | |  |  |  |  |  |
|  | Not in union | Ref |  |  |  |  |
|  | In union | 0.033 | 0.106 | 0.004 | **0.004** | **3.34** |
| **Place of residence** | |  |  |  |  |  |
|  | Urban | Ref |  |  |  |  |
|  | Rural | -0.299 | 0.080 | -0.024 | **-0.024** | **-20.40** |
| **Educational status** | |  |  |  |  |  |
|  | No education | Ref |  |  |  |  |
|  | Primary education | 0.083 | -0.154 | -0.013 | **0.022** | -9.04 |
|  | Secondary education | 0.137 | 0.238 | 0.033 |  | 25.00 |
|  | Higher education | 0.022 | 0.090 | 0.002 |  | 1.67 |
|  |  |  |  |  |  | **17.63** |
| **Wealth index** | |  |  |  |  |  |
|  | Poorest | Ref |  |  |  |  |
|  | Poorer | 0.043 | -0.307 | -0.013 | **0.071** | -9.90 |
|  | Middle | 0.048 | 0.044 | 0.002 |  | 1.59 |
|  | Richer | 0.077 | 0.381 | 0.029 |  | 23.39 |
|  | Richest | 0.096 | 0.552 | 0.053 |  | 43.31 |
|  |  |  |  |  |  | **58.39** |
| **Parity at the time of index pregnancy** | |  |  |  |  |  |
|  | 0 | Ref |  |  |  |  |
|  | 1 -2 | -0.055 | 0.031 | -0.002 | **0.001** | -1.49 |
|  | 3 - 4 | -0.040 | -0.010 | 0.000 |  | 0.36 |
|  | 5 - 6 | -0.032 | -0.049 | 0.002 |  | 1.32 |
|  | ≥ 7 | -0.029 | -0.036 | 0.001 |  | 0.90 |
|  |  |  |  |  |  | **1.10** |
| **Region within country** | |  |  |  |  |  |
|  | Cabinda | Ref |  |  |  |  |
|  | Zaire | 0.004 | -0.009 | 0.000 | **-0.039** | -0.034 |
|  | Uíge | -0.014 | -0.007 | 0.000 |  | 0.086 |
|  | Luanda | -0.111 | 0.374 | -0.041 |  | -35.317 |
|  | Cuanza Norte | -0.003 | -0.003 | 0.000 |  | 0.006 |
|  | Cuanza Sul | -0.044 | -0.015 | 0.001 |  | 0.572 |
|  | Malanje | -0.002 | 0.003 | 0.000 |  | -0.006 |
|  | Lunda Norte | -0.007 | -0.041 | 0.000 |  | 0.234 |
|  | Benguela | 0.009 | -0.072 | -0.001 |  | -0.581 |
|  | Huambo | -0.003 | -0.005 | 0.000 |  | 0.011 |
|  | Bié | -0.027 | -0.043 | 0.001 |  | 0.984 |
|  | Moxico | -0.010 | -0.012 | 0.000 |  | 0.098 |
|  | Cuando Cubango | -0.012 | -0.016 | 0.000 |  | 0.162 |
|  | Namibe | 0.003 | -0.005 | 0.000 |  | -0.012 |
|  | Huíla | -0.010 | -0.080 | 0.001 |  | 0.691 |
|  | Cunene | 0.000 | -0.072 | 0.000 |  | 0.014 |
|  | Lunda Sul | -0.005 | -0.019 | 0.000 |  | 0.078 |
|  | Bengo | -0.004 | 0.012 | 0.000 |  | -0.037 |
|  |  |  |  |  |  | **-33.05** |
| *Residual* | |  |  |  |  | ***51.12*** |

| **Decomposition analysis of inequality in primary maternal continuum of care in Benin** | | | | | | |
| --- | --- | --- | --- | --- | --- | --- |
| **Characteristics** | | **Elasticity** | **Conc. Index** | **Contribution** | **Total contribution** | **% contribution** |
| Age in years (at the time of the index birth) | |  |  |  |  |  |
|  | < 20 | Ref |  |  |  |  |
|  | 20-24 | 0.010 | 0.018 | 0.000 | **0.003** | 0.08 |
|  | 25-29 | 0.080 | 0.030 | 0.002 |  | 1.01 |
|  | 30-34 | 0.070 | 0.013 | 0.001 |  | 0.43 |
|  | 35-39 | 0.078 | -0.005 | 0.000 |  | -0.16 |
|  | 40-44 | 0.026 | -0.025 | 0.000 |  | -0.29 |
|  | 45-49 | 0.003 | -0.007 | 0.000 |  | -0.01 |
|  |  |  |  |  |  | **1.06** |
| Place of residence | |  |  |  |  |  |
|  | Urban | Ref |  |  |  |  |
|  | Rural | -0.131 | 0.025 | -0.003 | **-0.003** | **-1.38** |
| Educational status | |  |  |  |  |  |
|  | No education |  |  |  |  |  |
|  | Primary education | 0.055 | 0.110 | 0.006 | **0.013** | 2.61 |
|  | Secondary education | 0.033 | 0.209 | 0.007 |  | 2.95 |
|  | Higher education | 0.000 | 0.042 | 0.000 |  | -0.01 |
|  |  |  |  |  |  | **5.55** |
| Wealth index | |  |  |  |  |  |
|  | Poorest | Ref |  |  |  |  |
|  | Poorer | 0.109 | -0.313 | -0.034 | **0.145** | -14.56 |
|  | Middle | 0.165 | 0.006 | 0.001 |  | 0.40 |
|  | Richer | 0.185 | 0.334 | 0.062 |  | 26.42 |
|  | Richest | 0.187 | 0.622 | 0.116 |  | 49.76 |
|  |  |  |  |  |  | **62.01** |
| Parity at the time of index pregnancy | |  |  |  |  |  |
|  | 0 | Ref |  |  |  |  |
|  | 1 -2 | -0.077 | 0.101 | -0.008 | **-0.002** | -3.35 |
|  | 3 - 4 | -0.089 | -0.018 | 0.002 |  | 0.69 |
|  | 5 - 6 | -0.071 | -0.088 | 0.004 |  | 2.70 |
|  | ≥ 7 | -0.060 | -0.063 | 0.001 |  | 1.64 |
|  |  |  |  |  |  | **1.69** |
| *Residual* | |  |  |  |  | ***31.07*** |
|  |  |  |  |  |  |  |

| **Decomposition analysis of inequality in primary maternal continuum of care in Burundi** | | | | | | |
| --- | --- | --- | --- | --- | --- | --- |
| **Characteristics** | | **Elasticity** | **Conc. Index** | **Contribution** | **Total contribution** | **% contribution** |
| Age in years (at the time of the index birth) | |  |  |  |  |  |
|  | < 20 | Ref |  |  |  |  |
|  | 20-24 | 0.032 | -0.058 | -0.002 | **0.003** | -1.81 |
|  | 25-29 | 0.046 | 0.016 | 0.001 |  | 0.72 |
|  | 30-34 | 0.056 | 0.039 | 0.002 |  | 2.12 |
|  | 35-39 | 0.025 | 0.016 | 0.000 |  | 0.37 |
|  | 40-44 | 0.008 | 0.005 | 0.000 |  | 0.04 |
|  | 45-49 | -0.002 | 0.002 | 0.000 |  | 0.00 |
|  |  |  |  |  |  | **1.44** |
| Place of residence | |  |  |  |  |  |
|  | Urban | Ref |  |  |  |  |
|  | Rural | -0.830 | 0.007 | -0.006 | **-0.006** | **-5.67** |
| Educational status | |  |  |  |  |  |
|  | No education | Ref |  |  |  |  |
|  | Primary education | 0.058 | 0.064 | 0.004 | **0.012** | 3.52 |
|  | Secondary education | 0.046 | 0.171 | 0.008 |  | 7.53 |
|  | Higher education | 0.003 | 0.022 | 0.000 |  | 0.07 |
|  |  |  |  |  |  | **11.13** |
| Wealth index | |  |  |  |  |  |
|  | Poorest | Ref |  |  |  |  |
|  | Poorer | 0.049 | -0.285 | -0.014 | **0.056** | -13.32 |
|  | Middle | 0.031 | 0.067 | 0.002 |  | 1.99 |
|  | Richer | 0.059 | 0.354 | 0.021 |  | 19.96 |
|  | Richest | 0.083 | 0.564 | 0.047 |  | 44.57 |
|  |  |  |  |  |  | **53.20** |
| Parity at the time of index pregnancy | |  |  |  |  |  |
|  | 0 | Ref |  |  |  |  |
|  | 1 -2 | -0.087 | -0.034 | 0.003 | **0.001** | 2.86 |
|  | 3 - 4 | -0.075 | 0.017 | -0.001 |  | -1.19 |
|  | 5 - 6 | -0.050 | 0.007 | 0.000 |  | -0.35 |
|  | ≥ 7 | -0.041 | 0.012 | -0.001 |  | -0.48 |
|  |  |  |  |  |  | **0.83** |
| *Residual* | |  |  |  |  | ***39.08*** |
|  |  |  |  |  |  |  |

| **Decomposition analysis of inequality in primary maternal continuum of care in Cameroon** | | | | | | |
| --- | --- | --- | --- | --- | --- | --- |
| **Characteristics** | | **Elasticity** | **Conc. Index** | **Contribution** | **Total contribution** | **% contribution** |
| Age in years (at the time of the index birth) | |  |  |  |  |  |
|  | < 20 | Ref |  |  |  |  |
|  | 20-24 | 0.153 | 0.007 | 0.001 | **0.006** | 0.47 |
|  | 25-29 | 0.191 | 0.006 | 0.001 |  | 0.51 |
|  | 30-34 | 0.157 | 0.024 | 0.004 |  | 1.64 |
|  | 35-39 | 0.073 | 0.000 | 0.000 |  | 0.00 |
|  | 40-44 | 0.027 | -0.006 | 0.000 |  | -0.07 |
|  | 45-49 | 0.002 | -0.002 | 0.000 |  | 0.00 |
|  |  |  |  |  |  | **2.54** |
| Place of residence | |  |  |  |  |  |
|  | Urban | Ref |  |  |  |  |
|  | Rural | -0.262 | 0.057 | -0.015 | **-0.015** | **-6.60** |
| Educational status | |  |  |  |  |  |
|  | No education | Ref |  |  |  |  |
|  | Primary education | 0.253 | -0.009 | -0.002 | **0.123** | -1.04 |
|  | Secondary education | 0.431 | 0.272 | 0.117 |  | 52.21 |
|  | Higher education | 0.066 | 0.124 | 0.008 |  | 3.61 |
|  |  |  |  |  |  | **54.79** |
| Wealth index | |  |  |  |  |  |
|  | Poorest | Ref |  |  |  |  |
|  | Poorer | 0.049 | -0.326 | -0.011 | **0.072** | -5.04 |
|  | Middle | 0.057 | 0.019 | 0.001 |  | 0.49 |
|  | Richer | 0.058 | 0.342 | 0.020 |  | 8.77 |
|  | Richest | 0.103 | 0.604 | 0.062 |  | 27.65 |
|  |  |  |  |  |  | **31.87** |
| Parity at the time of index pregnancy | |  |  |  |  |  |
|  | 0 | Ref |  |  |  |  |
|  | 1 -2 | 0.112 | 0.065 | 0.007 | **0.006** | 3.22 |
|  | 3 - 4 | 0.020 | -0.036 | -0.001 |  | -0.32 |
|  | 5 - 6 | 0.020 | -0.033 | -0.001 |  | -0.30 |
|  | ≥ 7 | 0.004 | -0.061 | 0.000 |  | -0.12 |
|  |  |  |  |  |  | **2.49** |
| *Residual* | |  |  |  |  | ***14.91*** |
|  |  |  |  |  |  |  |

| **Decomposition analysis of inequality in primary maternal continuum of care in Ethiopia** | | | | | | |
| --- | --- | --- | --- | --- | --- | --- |
| **Characteristics** | | **Elasticity** | **Conc. Index** | **Contribution** | **Total contribution** | **% contribution** |
| Age in years (at the time of the index birth) | |  |  |  |  |  |
|  | < 20 | Ref |  |  |  |  |
|  | 20-24 | 0.003 | -0.026 | 0.000 | **0.001** | -0.09 |
|  | 25-29 | 0.047 | -0.006 | 0.000 |  | -0.31 |
|  | 30-34 | 0.043 | 0.037 | 0.002 |  | 1.74 |
|  | 35-39 | 0.028 | 0.007 | 0.000 |  | 0.21 |
|  | 40-44 | 0.013 | -0.001 | 0.000 |  | -0.01 |
|  | 45-49 | 0.002 | 0.002 | 0.000 |  | 0.00 |
|  |  |  |  |  |  | **1.54** |
| Place of residence | |  |  |  |  |  |
|  | Urban | Ref |  |  |  |  |
|  | Rural | -0.484 | -0.001 | 0.000 | **0.000** | **0.33** |
| Educational status | |  |  |  |  |  |
|  | No education | Ref |  |  |  |  |
|  | Primary education | 0.052 | 0.128 | 0.007 | **0.008** | 7.27 |
|  | Secondary education | 0.018 | 0.060 | 0.001 |  | 1.16 |
|  | Higher education | 0.012 | 0.056 | 0.001 |  | 0.75 |
|  |  |  |  |  |  | **9.18** |
| Wealth index | |  |  |  |  |  |
|  | Poorest | Ref |  |  |  |  |
|  | Poorer | 0.029 | -0.308 | -0.009 | **0.044** | -9.75 |
|  | Middle | 0.051 | 0.048 | 0.002 |  | 2.65 |
|  | Richer | 0.039 | 0.350 | 0.014 |  | 14.73 |
|  | Richest | 0.064 | 0.578 | 0.037 |  | 40.30 |
|  |  |  |  |  |  | **47.94** |
| Parity at the time of index pregnancy | |  |  |  |  |  |
|  | 0 | Ref |  |  |  |  |
|  | 1 -2 | -0.016 | 0.018 | 0.000 | **0.001** | -0.33 |
|  | 3 - 4 | -0.052 | -0.044 | 0.002 |  | 2.49 |
|  | 5 - 6 | -0.047 | -0.023 | 0.001 |  | 1.20 |
|  | ≥ 7 | -0.052 | 0.004 | 0.000 |  | -0.24 |
|  |  |  |  |  |  | **3.11** |
| *Residual* | |  |  |  |  | ***37.90*** |
|  |  |  |  |  |  |  |

| **Decomposition analysis of inequality in primary maternal continuum of care in Gambia** | | | | | | |
| --- | --- | --- | --- | --- | --- | --- |
| **Characteristics** | | **Elasticity** | **Conc. Index** | **Contribution** | **Total contribution** | **% contribution** |
| Age in years (at the time of the index birth) | |  |  |  |  |  |
|  | < 20 | Ref |  |  |  |  |
|  | 20-24 | -0.031 | -0.021 | 0.001 | **0.003** | 0.47 |
|  | 25-29 | 0.034 | 0.050 | 0.002 |  | 1.26 |
|  | 30-34 | 0.038 | 0.050 | 0.002 |  | 1.40 |
|  | 35-39 | 0.030 | -0.018 | -0.001 |  | -0.41 |
|  | 40-44 | 0.025 | -0.011 | 0.000 |  | -0.20 |
|  | 45-49 | 0.004 | -0.007 | 0.000 |  | -0.02 |
|  |  |  |  |  |  | **2.49** |
| Place of residence | |  |  |  |  |  |
|  | Urban | Ref |  |  |  |  |
|  | Rural | -0.093 | 0.012 | -0.001 | **-0.001** | **-0.80** |
| Educational status | |  |  |  |  |  |
|  | No education | Ref |  |  |  |  |
|  | Primary education | 0.008 | -0.009 | 0.000 | **0.010** | -0.05 |
|  | Secondary education | 0.057 | 0.179 | 0.010 |  | 7.54 |
|  | Higher education | 0.000 | 0.094 | 0.000 |  | -0.01 |
|  |  |  |  |  |  | **7.48** |
| Wealth index | |  |  |  |  |  |
|  | Poorest | Ref |  |  |  |  |
|  | Poorer | 0.038 | -0.310 | -0.012 | **0.089** | -8.64 |
|  | Middle | 0.056 | 0.038 | 0.002 |  | 1.54 |
|  | Richer | 0.076 | 0.332 | 0.025 |  | 18.49 |
|  | Richest | 0.121 | 0.607 | 0.073 |  | 53.77 |
|  |  |  |  |  |  | **65.17** |
| Parity at the time of index pregnancy | |  |  |  |  |  |
|  | 0 | Ref |  |  |  |  |
|  | 1 -2 | -0.039 | 0.062 | -0.002 | **0.004** | -1.76 |
|  | 3 - 4 | -0.096 | -0.024 | 0.002 |  | 1.70 |
|  | 5 - 6 | -0.047 | -0.040 | 0.002 |  | 1.38 |
|  | ≥ 7 | -0.036 | -0.051 | 0.002 |  | 1.34 |
|  |  |  |  |  |  | **2.66** |
| *Residual* | |  |  |  |  | ***23.00*** |
|  |  |  |  |  |  |  |

| **Decomposition analysis of inequality in primary maternal continuum of care in Guinea** | | | | | | |
| --- | --- | --- | --- | --- | --- | --- |
| **Characteristics** | | **Elasticity** | **Conc. Index** | **Contribution** | **Total contribution** | **% contribution** |
| Age in years (at the time of the index birth) | |  |  |  |  |  |
|  | < 20 | Ref |  |  |  |  |
|  | 20-24 | 0.040 | 0.009 | 0.000 | **0.001** | 0.25 |
|  | 25-29 | 0.030 | -0.004 | 0.000 |  | -0.08 |
|  | 30-34 | 0.046 | 0.014 | 0.001 |  | 0.44 |
|  | 35-39 | 0.021 | -0.005 | 0.000 |  | -0.07 |
|  | 40-44 | 0.020 | -0.006 | 0.000 |  | -0.08 |
|  | 45-49 | 0.007 | -0.005 | 0.000 |  | -0.03 |
|  |  |  |  |  |  | **0.43** |
| Place of residence | |  |  |  |  |  |
|  | Urban | Ref |  |  |  |  |
|  | Rural | -0.865 | 0.029 | -0.025 | **-0.025** | **-17.16** |
| Educational status | |  |  |  |  |  |
|  | No education | Ref |  |  |  |  |
|  | Primary education | 0.023 | 0.014 | 0.000 | **0.006** | 0.22 |
|  | Secondary education | 0.052 | 0.109 | 0.006 |  | 3.83 |
|  | Higher education | 0.008 | 0.040 | 0.000 |  | 0.21 |
|  |  |  |  |  |  | **4.26** |
| Wealth index | |  |  |  |  |  |
|  | Poorest | Ref |  |  |  |  |
|  | Poorer | 0.021 | -0.291 | -0.006 | **0.095** | -4.03 |
|  | Middle | 0.062 | 0.053 | 0.003 |  | 2.21 |
|  | Richer | 0.075 | 0.350 | 0.026 |  | 17.82 |
|  | Richest | 0.124 | 0.576 | 0.071 |  | 48.18 |
|  |  |  |  |  |  | **64.17** |
| Parity at the time of index pregnancy | |  |  |  |  |  |
|  | 0 | Ref |  |  |  |  |
|  | 1 -2 | -0.125 | -0.007 | 0.001 | **0.003** | 0.61 |
|  | 3 - 4 | -0.089 | -0.003 | 0.000 |  | 0.18 |
|  | 5 - 6 | -0.049 | -0.017 | 0.001 |  | 0.57 |
|  | ≥ 7 | -0.036 | -0.017 | 0.001 |  | 0.42 |
|  |  |  |  |  |  | **1.79** |
| *Residual* | |  |  |  |  | ***46.50*** |
|  |  |  |  |  |  |  |

| **Decomposition analysis of inequality in primary maternal continuum of care in Liberia** | | | | | | |
| --- | --- | --- | --- | --- | --- | --- |
| **Characteristics** | | **Elasticity** | **Conc. Index** | **Contribution** | **Total contribution** | **% contribution** |
| Age in years (at the time of the index birth) | |  |  |  |  |  |
|  | < 20 | Ref |  |  |  |  |
|  | 20-24 | 0.008 | 0.046 | 0.000 | **0.000** | 0.80 |
|  | 25-29 | -0.030 | 0.019 | -0.001 |  | -1.25 |
|  | 30-34 | -0.014 | -0.023 | 0.000 |  | 0.71 |
|  | 35-39 | 0.001 | -0.018 | 0.000 |  | -0.03 |
|  | 40-44 | -0.003 | -0.009 | 0.000 |  | 0.06 |
|  | 45-49 | 0.001 | -0.005 | 0.000 |  | -0.01 |
|  |  |  |  |  |  | **0.29** |
| Place of residence | |  |  |  |  |  |
|  | Urban | Ref |  |  |  |  |
|  | Rural | -0.056 | 0.012 | -0.001 | **-0.001** | **-1.46** |
| Educational status | |  |  |  |  |  |
|  | No education | Ref |  |  |  |  |
|  | Primary education | -0.008 | -0.114 | 0.001 | **0.016** | 2.06 |
|  | Secondary education | 0.064 | 0.217 | 0.014 |  | 30.50 |
|  | Higher education | 0.017 | 0.100 | 0.002 |  | 3.85 |
|  |  |  |  |  |  | **36.40** |
| Wealth index | |  |  |  |  |  |
|  | Poorest | Ref |  |  |  |  |
|  | Poorer | -0.046 | -0.299 | 0.014 | **0.021** | 30.72 |
|  | Middle | -0.011 | 0.042 | 0.000 |  | -1.02 |
|  | Richer | -0.007 | 0.349 | -0.002 |  | -5.48 |
|  | Richest | 0.017 | 0.582 | 0.010 |  | 21.39 |
|  |  |  |  |  |  | **45.61** |
| Parity at the time of index pregnancy | |  |  |  |  |  |
|  | 0 | Ref |  |  |  |  |
|  | 1 -2 | 0.053 | 0.069 | 0.004 | **0.001** | 8.08 |
|  | 3 - 4 | 0.027 | -0.073 | -0.002 |  | -4.31 |
|  | 5 - 6 | 0.009 | -0.061 | -0.001 |  | -1.23 |
|  | ≥ 7 | -0.001 | -0.021 | 0.000 |  | 0.04 |
|  |  |  |  |  |  | **2.58** |
| *Residual* | |  |  |  |  | ***16.58*** |
|  |  |  |  |  |  |  |

| **Decomposition analysis of inequality in primary maternal continuum of care in Malawi** | | | | | | |
| --- | --- | --- | --- | --- | --- | --- |
| **Characteristics** | | **Elasticity** | **Conc. Index** | **Contribution** | **Total contribution** | **% contribution** |
| Age in years (at the time of the index birth) | |  |  |  |  |  |
|  | < 20 | Ref |  |  |  |  |
|  | 20-24 | 0.040 | -0.013 | -0.001 | **0.004** | -0.82 |
|  | 25-29 | 0.046 | 0.043 | 0.002 |  | 3.09 |
|  | 30-34 | 0.050 | 0.035 | 0.002 |  | 2.74 |
|  | 35-39 | 0.040 | 0.008 | 0.000 |  | 0.53 |
|  | 40-44 | 0.013 | -0.002 | 0.000 |  | -0.04 |
|  | 45-49 | 0.002 | -0.001 | 0.000 |  | 0.00 |
|  |  |  |  |  |  | **5.49** |
| Place of residence | |  |  |  |  |  |
|  | Urban | Ref |  |  |  |  |
|  | Rural | -0.214 | -0.003 | 0.001 | **0.001** | **0.96** |
| Educational status | |  |  |  |  |  |
|  | No education | Ref |  |  |  |  |
|  | Primary education | 0.109 | -0.172 | -0.019 | **-0.003** | -29.27 |
|  | Secondary education | 0.064 | 0.218 | 0.014 |  | 22.02 |
|  | Higher education | 0.021 | 0.057 | 0.001 |  | 1.83 |
|  |  |  |  |  |  | **-5.42** |
| Wealth index | |  |  |  |  |  |
|  | Poorest | Ref |  |  |  |  |
|  | Poorer | -0.008 | -0.268 | 0.002 | **0.020** | 3.53 |
|  | Middle | -0.001 | 0.089 | 0.000 |  | -0.12 |
|  | Richer | 0.001 | 0.367 | 0.000 |  | 0.71 |
|  | Richest | 0.033 | 0.536 | 0.018 |  | 27.66 |
|  |  |  |  |  |  | **31.78** |
| Parity at the time of index pregnancy | |  |  |  |  |  |
|  | 0 | Ref |  |  |  |  |
|  | 1 -2 | -0.019 | 0.025 | 0.000 | **0.001** | -0.76 |
|  | 3 - 4 | -0.046 | -0.008 | 0.000 |  | 0.59 |
|  | 5 - 6 | -0.045 | -0.012 | 0.001 |  | 0.85 |
|  | ≥ 7 | -0.015 | -0.010 | 0.000 |  | 0.24 |
|  |  |  |  |  |  | **0.92** |
| *Residual* | |  |  |  |  | ***66.27*** |
|  |  |  |  |  |  |  |

| **Decomposition analysis of inequality in primary maternal continuum of care in Mali** | | | | | | |
| --- | --- | --- | --- | --- | --- | --- |
| **Characteristics** | | **Elasticity** | **Conc. Index** | **Contribution** | **Total contribution** | **% contribution** |
| Age in years (at the time of the index birth) | |  |  |  |  |  |
|  | < 20 | Ref |  |  |  |  |
|  | 20-24 | 0.016 | 0.072 | 0.001 | **-0.002** | 0.54 |
|  | 25-29 | 0.050 | -0.003 | 0.000 |  | -0.07 |
|  | 30-34 | 0.055 | -0.017 | -0.001 |  | -0.44 |
|  | 35-39 | 0.032 | -0.058 | -0.002 |  | -0.88 |
|  | 40-44 | 0.006 | -0.031 | 0.000 |  | -0.09 |
|  | 45-49 | 0.003 | -0.006 | 0.000 |  | -0.01 |
|  |  |  |  |  |  | **-0.94** |
| Place of residence | |  |  |  |  |  |
|  | Urban | Ref |  |  |  |  |
|  | Rural | -0.752 | 0.021 | -0.015 | **-0.015** | **-7.28** |
| Educational status | |  |  |  |  |  |
|  | No education | Ref |  |  |  |  |
|  | Primary education | 0.057 | 0.040 | 0.002 | **0.020** | 1.06 |
|  | Secondary education | 0.087 | 0.199 | 0.017 |  | 8.14 |
|  | Higher education | 0.003 | 0.036 | 0.000 |  | 0.05 |
|  |  |  |  |  |  | **9.25** |
| Wealth index | |  |  |  |  |  |
|  | Poorest | Ref |  |  |  |  |
|  | Poorer | 0.067 | -0.337 | -0.022 | **0.140** | -10.57 |
|  | Middle | 0.092 | -0.006 | -0.001 |  | -0.26 |
|  | Richer | 0.107 | 0.330 | 0.035 |  | 16.56 |
|  | Richest | 0.203 | 0.629 | 0.127 |  | 59.89 |
|  |  |  |  |  |  | **65.62** |
| Parity at the time of index pregnancy | |  |  |  |  |  |
|  | 0 | Ref |  |  |  |  |
|  | 1 -2 | -0.062 | 0.106 | -0.007 | **0.000** | -3.06 |
|  | 3 - 4 | -0.072 | -0.030 | 0.002 |  | 1.01 |
|  | 5 - 6 | -0.043 | -0.055 | 0.002 |  | 1.11 |
|  | ≥ 7 | -0.020 | -0.113 | 0.002 |  | 1.05 |
|  |  |  |  |  |  | **0.11** |
| *Residual* | |  |  |  |  | ***33.24*** |
|  |  |  |  |  |  |  |

| **Decomposition analysis of inequality in primary maternal continuum of care in Nigeria** | | | | | | |
| --- | --- | --- | --- | --- | --- | --- |
| **Characteristics** | | **Elasticity** | **Conc. Index** | **Contribution** | **Total contribution** | **% contribution** |
| Age in years (at the time of the index birth) | |  |  |  |  |  |
|  | < 20 | Ref |  |  |  |  |
|  | 20-24 | 0.032 | -0.024 | -0.001 | **0.008** | -0.24 |
|  | 25-29 | 0.081 | 0.045 | 0.004 |  | 1.13 |
|  | 30-34 | 0.110 | 0.048 | 0.005 |  | 1.63 |
|  | 35-39 | 0.078 | 0.011 | 0.001 |  | 0.26 |
|  | 40-44 | 0.036 | -0.017 | -0.001 |  | -0.20 |
|  | 45-49 | 0.006 | -0.003 | 0.000 |  | -0.01 |
|  |  |  |  |  |  | **2.59** |
| Place of residence | |  |  |  |  |  |
|  | Urban | Ref |  |  |  |  |
|  | Rural | -0.352 | -0.005 | 0.002 | **0.002** | **0.51** |
| Educational status | |  |  |  |  |  |
|  | No education | Ref |  |  |  |  |
|  | Primary education | 0.089 | -0.014 | -0.001 | **0.097** | -0.38 |
|  | Secondary education | 0.267 | 0.294 | 0.078 |  | 24.26 |
|  | Higher education | 0.103 | 0.196 | 0.020 |  | 6.29 |
|  |  |  |  |  |  | **30.16** |
| Wealth index | |  |  |  |  |  |
|  | Poorest | Ref |  |  |  |  |
|  | Poorer | 0.050 | -0.324 | -0.016 | **0.105** | -4.99 |
|  | Middle | 0.087 | 0.031 | 0.003 |  | 0.83 |
|  | Richer | 0.113 | 0.365 | 0.041 |  | 12.77 |
|  | Richest | 0.135 | 0.574 | 0.077 |  | 23.98 |
|  |  |  |  |  |  | **32.59** |
| Parity at the time of index pregnancy | |  |  |  |  |  |
|  | 0 | Ref |  |  |  |  |
|  | 1 -2 | -0.088 | 0.120 | -0.011 | **0.006** | -3.27 |
|  | 3 - 4 | -0.102 | -0.009 | 0.001 |  | 0.29 |
|  | 5 - 6 | -0.081 | -0.062 | 0.005 |  | 1.56 |
|  | ≥ 7 | -0.099 | -0.106 | 0.011 |  | 3.27 |
|  |  |  |  |  |  | **1.85** |
| *Residual* | |  |  |  |  | ***32.29*** |

| **Decomposition analysis of inequality in primary maternal continuum of care in Sierra Leone** | | | | | | |
| --- | --- | --- | --- | --- | --- | --- |
| **Characteristics** | | **Elasticity** | **Conc. Index** | **Contribution** | **Total contribution** | **% contribution** |
| Age in years (at the time of the index birth) | |  |  |  |  |  |
|  | < 20 | Ref |  |  |  |  |
|  | 20-24 | -0.054 | 0.041 | -0.002 | **-0.001** | -5.38 |
|  | 25-29 | -0.042 | 0.015 | -0.001 |  | -1.56 |
|  | 30-34 | -0.046 | -0.011 | 0.001 |  | 1.22 |
|  | 35-39 | -0.024 | -0.031 | 0.001 |  | 1.79 |
|  | 40-44 | -0.008 | -0.017 | 0.000 |  | 0.35 |
|  | 45-49 | -0.001 | -0.003 | 0.000 |  | 0.01 |
|  |  |  |  |  |  | **-3.57** |
| Place of residence | |  |  |  |  |  |
|  | Urban | Ref |  |  |  |  |
|  | Rural | 0.053 | 0.019 | 0.001 | **0.001** | **2.47** |
| Educational status | |  |  |  |  |  |
|  | No education | Ref |  |  |  |  |
|  | Primary education | 0.018 | -0.010 | 0.000 | **0.008** | -0.46 |
|  | Secondary education | 0.054 | 0.138 | 0.007 |  | 17.95 |
|  | Higher education | 0.014 | 0.061 | 0.001 |  | 2.11 |
|  |  |  |  |  |  | **19.59** |
| Wealth index | |  |  |  |  |  |
|  | Poorest | Ref |  |  |  |  |
|  | Poorer | -0.029 | -0.315 | 0.009 | **0.024** | 22.18 |
|  | Middle | 0.000 | 0.033 | 0.000 |  | -0.01 |
|  | Richer | 0.024 | 0.362 | 0.009 |  | 21.19 |
|  | Richest | 0.011 | 0.579 | 0.006 |  | 15.15 |
|  |  |  |  |  |  | **58.51** |
| Parity at the time of index pregnancy | |  |  |  |  |  |
|  | 0 | Ref |  |  |  |  |
|  | 1 -2 | 0.006 | 0.068 | 0.000 | **0.006** | 1.01 |
|  | 3 - 4 | 0.027 | -0.053 | -0.001 |  | -3.42 |
|  | 5 - 6 | 0.007 | -0.046 | 0.000 |  | -0.74 |
|  | ≥ 7 | 0.003 | -0.034 | 0.000 |  | -0.25 |
|  |  |  |  |  |  | **-3.40** |
| *Residual* | |  |  |  |  | ***26.40*** |

| **Decomposition analysis of inequality in primary maternal continuum of care in South Africa** | | | | | | |
| --- | --- | --- | --- | --- | --- | --- |
| **Characteristics** | | **Elasticity** | **Conc. Index** | **Contribution** | **Total contribution** | **% contribution** |
| Age in years (at the time of the index birth) | |  |  |  |  |  |
|  | < 20 | Ref |  |  |  |  |
|  | 20-24 | 0.012 | -0.051 | -0.001 | **0.000** | -1.18 |
|  | 25-29 | -0.002 | 0.033 | 0.000 |  | -0.12 |
|  | 30-34 | 0.013 | 0.043 | 0.001 |  | 1.11 |
|  | 35-39 | -0.004 | 0.016 | 0.000 |  | -0.12 |
|  | 40-44 | 0.011 | 0.005 | 0.000 |  | 0.11 |
|  | 45-49 | -0.002 | 0.000 | 0.000 |  | 0.00 |
|  |  |  |  |  |  | **-0.20** |
| Place of residence | |  |  |  |  |  |
|  | Urban | Ref |  |  |  |  |
|  | Rural | -0.011 | 0.134 | -0.001 | **-0.001** | **-2.82** |
| Educational status | |  |  |  |  |  |
|  | No education | Ref |  |  |  |  |
|  | Primary education | -0.008 | -0.130 | 0.001 | **0.004** | 1.90 |
|  | Secondary education | 0.157 | -0.063 | -0.010 |  | -19.32 |
|  | Higher education | 0.061 | 0.212 | 0.013 |  | 25.33 |
|  |  |  |  |  |  | **7.91** |
| Wealth index | |  |  |  |  |  |
|  | Poorest | Ref |  |  |  |  |
|  | Poorer | 0.072 | -0.238 | -0.017 | **0.007** | -33.52 |
|  | Middle | 0.043 | 0.123 | 0.005 |  | 10.21 |
|  | Richer | 0.032 | 0.363 | 0.012 |  | 22.43 |
|  | Richest | 0.015 | 0.511 | 0.008 |  | 14.91 |
|  |  |  |  |  |  | **14.03** |
| Parity at the time of index pregnancy | |  |  |  |  |  |
|  | 0 | Ref |  |  |  |  |
|  | 1 -2 | -0.037 | 0.054 | -0.002 | **0.006** | -3.93 |
|  | 3 - 4 | -0.011 | -0.076 | 0.001 |  | 1.68 |
|  | 5 - 6 | -0.011 | -0.009 | 0.000 |  | 0.20 |
|  | ≥ 7 | -0.004 | -0.012 | 0.000 |  | 0.08 |
|  |  |  |  |  |  | **-1.97** |
| *Residual* | |  |  |  |  | ***83.06*** |

| **Decomposition analysis of inequality in primary maternal continuum of care in Tanzania** | | | | | | |
| --- | --- | --- | --- | --- | --- | --- |
| **Characteristics** | | **Elasticity** | **Conc. Index** | **Contribution** | **Total contribution** | **% contribution** |
| Age in years (at the time of the index birth) | |  |  |  |  |  |
|  | < 20 | Ref |  |  |  |  |
|  | 20-24 | 0.019 | 0.010 | 0.000 | **0.003** | 0.13 |
|  | 25-29 | 0.082 | 0.051 | 0.004 |  | 2.90 |
|  | 30-34 | 0.097 | 0.020 | 0.002 |  | 1.31 |
|  | 35-39 | 0.087 | -0.022 | -0.002 |  | -1.32 |
|  | 40-44 | 0.043 | -0.032 | -0.001 |  | -0.94 |
|  | 45-49 | 0.008 | 0.003 | 0.000 |  | 0.02 |
|  |  |  |  |  |  | **2.10** |
| Place of residence | |  |  |  |  |  |
|  | Urban | Ref |  |  |  |  |
|  | Rural | -0.410 | 0.000 | 0.000 | **0.048** | **0.05** |
| Educational status | |  |  |  |  |  |
|  | No education | Ref |  |  |  |  |
|  | Primary education | 0.170 | -0.039 | -0.007 | **0.007** | -4.56 |
|  | Secondary education | 0.062 | 0.221 | 0.014 |  | 9.47 |
|  | Higher education | 0.005 | 0.024 | 0.000 |  | 0.09 |
|  |  |  |  |  |  | **4.99** |
| Wealth index | |  |  |  |  |  |
|  | Poorest | Ref |  |  |  |  |
|  | Poorer | 0.049 | -0.301 | -0.015 | **0.060** | -10.28 |
|  | Middle | 0.051 | 0.042 | 0.002 |  | 1.50 |
|  | Richer | 0.065 | 0.355 | 0.023 |  | 15.89 |
|  | Richest | 0.085 | 0.581 | 0.049 |  | 34.21 |
|  |  |  |  |  |  | **41.32** |
| Parity at the time of index pregnancy | |  |  |  |  |  |
|  | 0 | Ref |  |  |  |  |
|  | 1 -2 | -0.123 | 0.050 | -0.006 | **0.011** | -4.24 |
|  | 3 - 4 | -0.129 | -0.020 | 0.003 |  | 1.78 |
|  | 5 - 6 | -0.127 | -0.052 | 0.007 |  | 4.60 |
|  | ≥ 7 | -0.108 | -0.075 | 0.008 |  | 5.59 |
|  |  |  |  |  |  | **7.74** |
| *Residual* | |  |  |  |  | ***43.81*** |

| **Decomposition analysis of inequality in primary maternal continuum of care in Uganda** | | | | | | |
| --- | --- | --- | --- | --- | --- | --- |
| **Characteristics** | | **Elasticity** | **Conc. Index** | **Contribution** | **Total contribution** | **% contribution** |
| Age in years (at the time of the index birth) | |  |  |  |  |  |
|  | < 20 | Ref |  |  |  |  |
|  | 20-24 | 0.036 | 0.027 | 0.001 | **0.002** | 0.74 |
|  | 25-29 | 0.058 | 0.043 | 0.002 |  | 1.87 |
|  | 30-34 | 0.060 | -0.007 | 0.000 |  | -0.30 |
|  | 35-39 | 0.043 | -0.012 | -0.001 |  | -0.39 |
|  | 40-44 | 0.020 | -0.007 | 0.000 |  | -0.11 |
|  | 45-49 | 0.002 | -0.005 | 0.000 |  | -0.01 |
|  |  |  |  |  |  | **1.81** |
| Place of residence | |  |  |  |  |  |
|  | Urban | Ref |  |  |  |  |
|  | Rural | -0.326 | -0.038 | 0.012 | **0.012** | **9.35** |
| Educational status | |  |  |  |  |  |
|  | No education | Ref |  |  |  |  |
|  | Primary education | -0.009 | -0.257 | 0.002 | **0.032** | 1.67 |
|  | Secondary education | 0.092 | 0.237 | 0.022 |  | 16.42 |
|  | Higher education | 0.053 | 0.147 | 0.008 |  | 5.83 |
|  |  |  |  |  |  | **23.92** |
| Wealth index | |  |  |  |  |  |
|  | Poorest | Ref |  |  |  |  |
|  | Poorer | -0.020 | -0.313 | 0.006 | **0.045** | 4.60 |
|  | Middle | -0.012 | 0.014 | 0.000 |  | -0.12 |
|  | Richer | 0.008 | 0.334 | 0.003 |  | 2.01 |
|  | Richest | 0.059 | 0.619 | 0.037 |  | 27.55 |
|  |  |  |  |  |  | **34.04** |
| Parity at the time of index pregnancy | |  |  |  |  |  |
|  | 0 | Ref |  |  |  |  |
|  | 1 -2 | -0.105 | 0.073 | -0.008 | **0.004** | -5.72 |
|  | 3 - 4 | -0.115 | -0.015 | 0.002 |  | 1.30 |
|  | 5 - 6 | -0.079 | -0.066 | 0.005 |  | 3.92 |
|  | ≥ 7 | -0.098 | -0.050 | 0.005 |  | 3.68 |
|  |  |  |  |  |  | **3.18** |
| *Residual* | |  |  |  |  | ***27.71*** |

| **Decomposition analysis of inequality in primary maternal continuum of care in Zambia** | | | | | | |
| --- | --- | --- | --- | --- | --- | --- |
| **Characteristics** | | **Elasticity** | **Conc. Index** | **Contribution** | **Total contribution** | **% contribution** |
| Age in years (at the time of the index birth) | |  |  |  |  |  |
|  | < 20 | Ref |  |  |  |  |
|  | 20-24 | 0.045 | -0.017 | -0.001 | **0.005** | -0.66 |
|  | 25-29 | 0.054 | 0.037 | 0.002 |  | 1.76 |
|  | 30-34 | 0.074 | 0.047 | 0.004 |  | 3.07 |
|  | 35-39 | 0.049 | 0.008 | 0.000 |  | 0.35 |
|  | 40-44 | 0.021 | -0.012 | 0.000 |  | -0.22 |
|  | 45-49 | 0.000 | 0.000 | 0.000 |  | 0.00 |
|  |  |  |  |  |  | **4.29** |
| Place of residence | |  |  |  |  |  |
|  | Urban | Ref |  |  |  |  |
|  | Rural | -0.433 | -0.002 | 0.001 | **0.001** | **0.64** |
| Educational status | |  |  |  |  |  |
|  | No education | Ref |  |  |  |  |
|  | Primary education | 0.127 | -0.242 | -0.031 | **0.016** | -26.65 |
|  | Secondary education | 0.235 | 0.012 | 0.045 |  | 39.03 |
|  | Higher education | 0.018 | 0.123 | 0.002 |  | 1.96 |
|  |  |  |  |  |  | **14.34** |
| Wealth index | |  |  |  |  |  |
|  | Poorest | Ref |  |  |  |  |
|  | Poorer | 0.051 | -0.282 | -0.014 | **0.047** | -12.51 |
|  | Middle | 0.051 | 0.079 | 0.004 |  | 3.51 |
|  | Richer | 0.036 | 0.359 | 0.013 |  | 11.38 |
|  | Richest | 0.081 | 0.551 | 0.045 |  | 38.94 |
|  |  |  |  |  |  | **41.31** |
| Parity at the time of index pregnancy | |  |  |  |  |  |
|  | 0 | Ref |  |  |  |  |
|  | 1 -2 | -0.059 | 0.033 | -0.002 | **0.002** | -1.70 |
|  | 3 - 4 | -0.078 | 0.004 | 0.000 |  | -0.30 |
|  | 5 - 6 | -0.070 | -0.046 | 0.003 |  | 2.78 |
|  | ≥ 7 | -0.040 | -0.017 | 0.001 |  | 0.62 |
|  |  |  |  |  |  | **1.41** |
| *Residual* | |  |  |  |  | ***38.02*** |
